# Designed Water Capture in Terpene Synthase Catalysis

**DOI:** 10.1002/cbic.70265

**Published:** 2026-03-12

**Authors:** Prabhakar L. Srivastava, David J. Miller, Rudolf K. Allemann

**Affiliations:** ^1^ School of Chemistry Cardiff University Cardiff UK

**Keywords:** enzyme engineering, protein engineering, solvation, terpene synthase

## Abstract

Sesquiterpene synthases catalyse cyclisations and rearrangements of farnesyl diphosphate to produce a diverse array of sesquiterpenes generated by depronotation and/or water capture. However, the precise mechanisms and dynamics controlling the fate of the final carbocationic intermediate are not well understood. In our previous study, we engineered water capture in selina‐4(15),7(11)‐diene synthase (SpSdS) to produce selin‐7(11)‐en‐4‐ol as a major product at pH 6.0 by point mutation (G305E) in the K_helix_ region. To develop a more generalised protocol for this functional switch in sesquiterpene synthases, we identified and characterised a novel selina‐3,7(11)‐diene synthase (AsSdS) from *Actinacidiphila soli* through multiple sequence alignments which naturally contains glutamate at position 305 (E305). Through site‐directed mutagenesis, creating variant G221T, we were able to instigate water capture in AsSdS to produce selin‐7(11)‐en‐4‐ol. Our findings identified two crucial regions in the active site pocket of selinadiene synthases: G/E305 in K_helix_ and T/G221 in H_helix_, that have a reproducible effect on product outcome determination. We demonstrate that subtle, yet predictable changes to these residues impact the water capture as well as deprotonation capability of selinadiene synthases and this solvation aspect can be further exploited to engineer other terpene synthases to generate biocatalysts with unique product profiles for diverse applications.

## Graphical abstract

Engineering of water capture in terpene synthases remains very challenging. We identified two regions in the selinadiene synthase active site pocket that can influence deprotonation or water capture in the final catalytic step and demonstrate a designed‐in switch in product outcome. These insights can be used for engineering other sesquiterpene synthases to generate new biocatalysts.

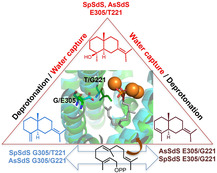

## 1 Introduction

Terpenoids or isoprenoids are the largest known family of secondary metabolites [1, 2]. To date, over 100,000 naturally occurring terpenoids have been reported and they collectively have numerous biological activities with industrial applications such as in flavour and fragrances, cosmetics and pharmaceuticals as well as in agriculture [1, 3, 4]. All these terpenoids are produced from two five carbon precursors dimethylallyl diphosphate (DMADP) and isopentenyl diphosphate (IDP). These are joined by head to tail condensation catalysed by prenyl transferases to form higher isoprenyl diphosphates such as geranyl diphosphate (GDP), farnesyl diphosphate (FDP) and geranylgeranyl diphosphate (GGDP). The first committed step in the formation of the diverse array of carbon skeletons is the cyclisation of acyclic isoprenyl diphosphates catalysed by terpene synthases (TSs) [4]. In Class 1 TSs, upon substrate binding, an allylic carbocation is formed by removal of diphosphate moiety triggered by Mg^2+^ coordination [3, 5, 6]. In the subsequent step, this highly reactive carbocation undergoes a series of intra‐molecular reactions such as: ring closures, hydride shifts, methyl and alkyl shifts before final products are formed by either deprotonation or quenching the reactive carbocation and/or neutral intermediate with trapped active site water [3, 7, 8, 9, 10, 11]. Even though TSs share a highly conserved active site architecture and follow a similar catalytic mechanism, minor changes in the amino acid composition can significantly affect the product outcomes [12, 13]. In TSs, the active site cavity is most often composed of six to eight alpha‐helical bundles, which undergo significant structural change after substrate binding from apo to closed confirmation [14, 15, 16, 17]. Despite extensive studies performed to understand the catalytic mechanism of TSs, limited insights exist into the regulation of highly reactive carbocations and their final deprotonation/ water capture [18, 19].

**SCHEME 1:**
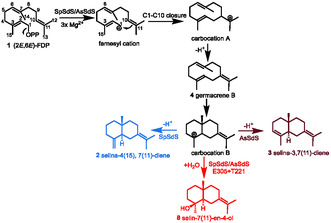
Cyclisation mechanism of products arising from selina‐4(15),7(11)‐diene synthase, selina‐3,7(11)‐diene synthases and their variants.

In previous work [20], we successfully engineered the non‐hydroxylating sesquiterpene synthase, selina‐4(15),7(11)‐diene synthase from *Streptomyces pristinaespiralis* ATCC 25486 (SpSdS) for utilising a trapped active site water molecule to quench the final reactive carbocation B, producing hydroxylated sesquiterpene alcohol (selin‐7(11)‐en‐4‐ol, **8**, Scheme 1). Molecular dynamic simulations carried out using SdS_WT_ and SdS G305E variant with carbocation B revealed that a high mobility water molecule was observed approaching C3 (FDP numbering) in the SdS G305E variant, which was coordinated by mostly PPi and sometimes by E305 to assist in quenching the carbocation [20]. Moreover, this quenching at C3 and/or deprotonation from C15 is subtle and might lead to the formation of a hydrocarbon product in the absence of a water molecule in the close vicinity to C3 and PPi. In addition to this, our previous work on engineering germacradiene‐11‐ol synthase (Gd11olS) had indicated the crucial role of the corresponding residue (H320) in water capture [19]. Whereas replacement of Y525 and Y531 (present at the J‐K loop) in patchoulol synthase (PTS) resulted in avoidance of water capture [11]. In aristolochene synthase from *Aspergillus terreus* (AtAS), mutating the corresponding (S303D) residue also resulted in the formation of linear hydroxylated products, highlighting the critical role of this residue in water capture [21].

**FIGURE 1:**
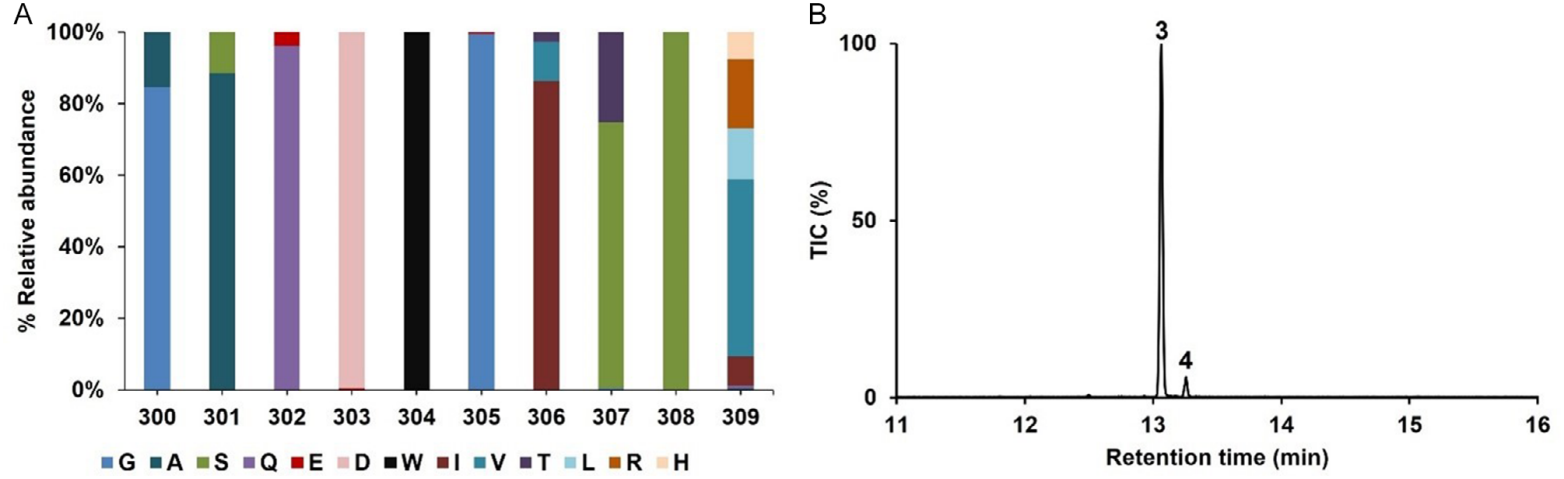
(A) The relative abundance and conservation of residues present at 300–309 in the K_helix_ region in all the potential selinadiene synthases (182 sequences). (B) Total ion chromatogram of the pentane soluble products arising from incubation of selina‐3,7(11)‐diene synthase from *Actinacidiphila soli* (AsSdS) with (2*E*,6*E*)‐FDP (**1**). Selina‐3,7(11)‐diene (**3**) is the major product along with a small amount of germacrene B (**4**).

**FIGURE 2:**
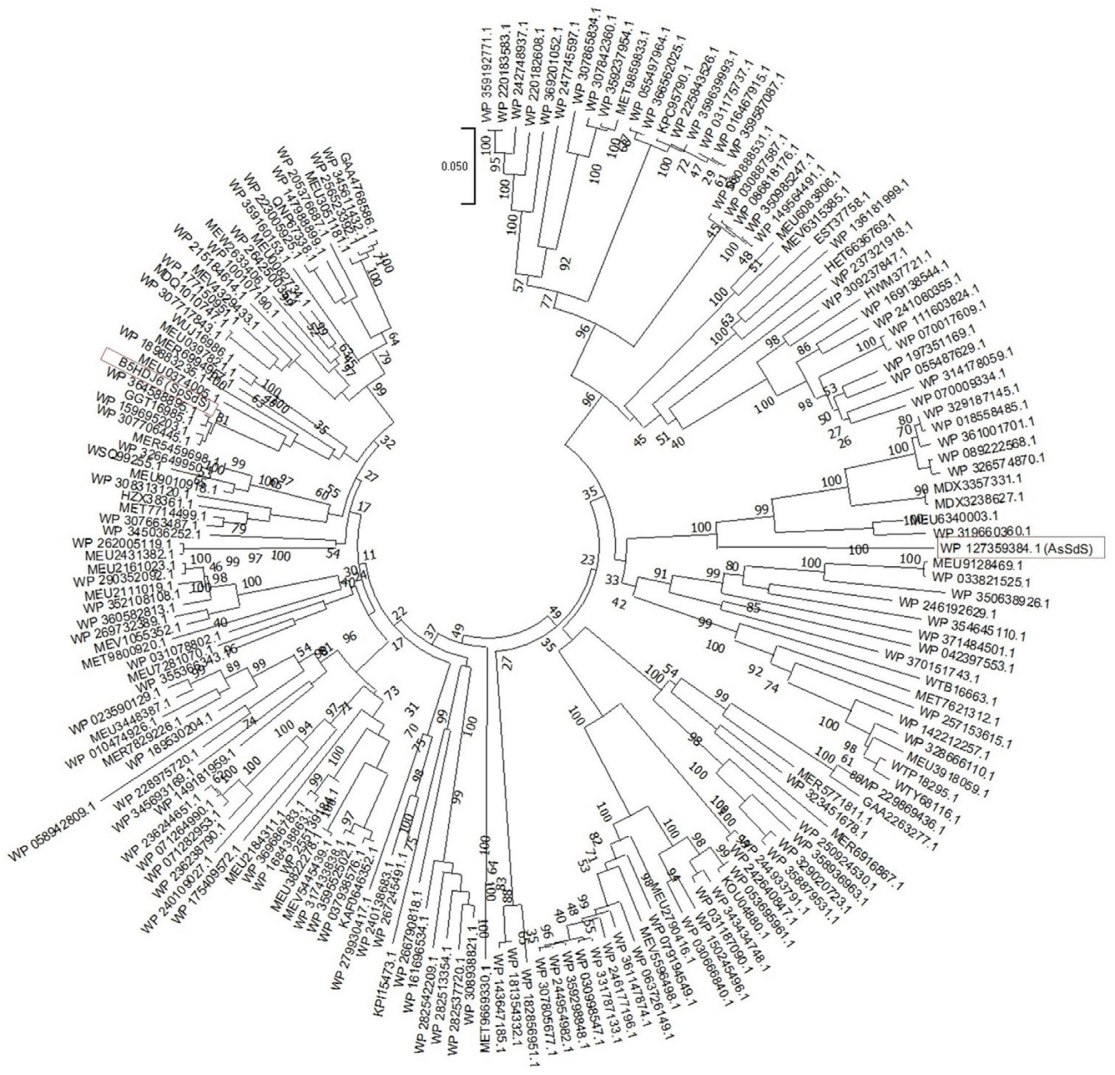
Phylogenetic tree for all the potential selinadiene synthases identified in this work showing a significant match, ≥70% identity at amino acid level, to SpSdS (B5HDJ6).

To establish a generalised approach to introduce water capture in sesquiterpene synthases, we carried out a BLAST search using the SpSdS amino acid (primary) sequence against the non‐redundant database, and 182 potential selinadiene synthases were selected with sequence identities of ≥70% (Figure S1). Multiple sequence alignment of all the potential selinadiene synthases revealed that they share a high level of conservation of key residues involved in catalysis. We focused our analysis on the sequence similarity of residues in the K_helix_ region (300–309), finding that D303, W304 and S308 are conserved in all homologues (Figure 1A). Additionally, G305 is also highly conserved except for one sequence reported from *Actinacidiphila soli* (AsSdS, WP_127359384), which contains E305 (Figure 1A, Figure S1). To investigate the role of E305 in AsSdS, the gene sequence was overexpressed in the pET28a vector, and the recombinant protein was purified to homogeneity (see Supporting Information for more details). Incubation of purified AsSdS with (2*E*,6*E*)‐FDP (**1**) resulted in the formation of selina‐3,7(11)‐diene (**3,** 93%) as a major product along with a small amount of germacrene B (**4**, 7%) (Figure 1B). We constructed a neighbour‐joining phylogenetic tree to establish the evolutionary relationship between these sequences using MEGA11 [22]. Phylogenetic analysis indicated that, despite a high level of sequence similarity, all the potential selinadiene synthases are divided into two major clades, one containing the SpSdS (B5HDJ6), while the sequence (AsSdS) containing E at 305 in K_helix_ is part of the other clade (Figure 2).

To determine the structure of the major sesquiterpene (**3**) produced by AsSdS, preparative scale incubation with **1** (26.8 mg) was performed (see Supporting Information for more details), which resulted in 6.5 mg of total product as a colourless liquid. The major compound was identified as selina‐3,7(11)‐diene using nuclear magnetic resonance (NMR) spectroscopy by comparison of the spectra with the data for genuine material previously reported [23, 24, 25] (spectroscopic assignment of all the ^1^H and ^13^C NMR spectra is given in the Supporting Information, Figures S23‐25). Formation of **3** follows a catalytic mechanism similar to that for **2**, only differing in the location of the final deprotonation. In brief, the Mg^2+^‐mediated diphosphate (PPi) cleavage of **1** followed by 1,10‐ring closure to form carbocation A. This undergoes deprotonation from C10 to form germacrene B (**4**). Following this, **4** gets protonated in a process mediated by G182 (*inter alia*) and undergoes a second ring closure (C2‐C7) to form carbocation B, which in turn, gets deprotonated from C4 and/or C15 to generate **3** and/or **2**, respectively (Scheme 1, FDP numbering). The kinetic parameters for AsSdS determined using 1‐^3^H labelled (2*E*,6*E*)‐FDP were *K*_M_ = 1.5 ± 0.6 µM, and *k*_cat_ = 6.0 ± 0.7 x 10^−3^ s^−1^ (Table 1), which are consistent with the range of kinetic constants reported for other terpene synthases [26, 27, 28].

**TABLE 1:** Kinetic parameters determined for AsSdS and SpSdS and their variants.

	*K*_M_ (μM)	*k*_cat_ (s^−1^) × 10^−3^	*k*_cat_/*K*_M_ (μM^−1^ s^−1^) × 10^−3^
AsSdS	1.5 ± 0.6	6.0 ± 0.7	4.0
AsSdS E305G	1.9 ± 0.6	6.0 ± 0.6	3.2
AsSdS G221T	0.5 ± 0.28	2.0 ± 0.3	4.0
SpSdS	0.86 ± 0.11	7.0 ± 0.02	8.14
SpSdS G305E	1.86 ± 0.19	4.0 ± 0.01	2.22
SpSdS G305E/T221G	0.8 ± 0.3	4.0 ± 0.3	5.0

Selina‐3,7(11)‐diene has been isolated from various sources and possesses numerous biological activities, including insect‐repellent activity, and has agrochemical potential via putative protection of high‐value commercial crops from insect infestation [24, 29]. However, little progress has been made on isolation and enzymatic characterisation of a sesquiterpene synthase for selina‐3,7(11)‐diene biosynthesis [30, 31]. Our previous report showed that, upon incorporation of E at 305 position in SpSdS (G305E), the product mixture contained 20% of **8** at pH 8.0, which, on further optimisation, was enhanced to 48% (as the major product) at pH 6.0 along with traces of **3** (6.3%) [20]. Selin‐7(11)‐en‐4‐ol (**8**) is formed by quenching of carbocation B with water at the C3 (FDP numbering, Scheme 1). Moreover, despite the presence of E at 305 in AsSdS, no selin‐7(11)‐en‐4‐ol (**8**) formation was observed in the assay mixture, indicating that G/E305 has a crucial role in final product determination. However, E305 may not alone be responsible for the formation of **8**, and several residues might have a coordinated effect by surrounding the reactive carbocation and affecting the water availability and activation. Therefore, this provides a perfect opportunity to delineate the cyclisation and water capture mechanisms in SpSdS and AsSdS, which share high sequence similarity and follow similar reaction mechanisms (Scheme 1 **)**.

**FIGURE 3:**
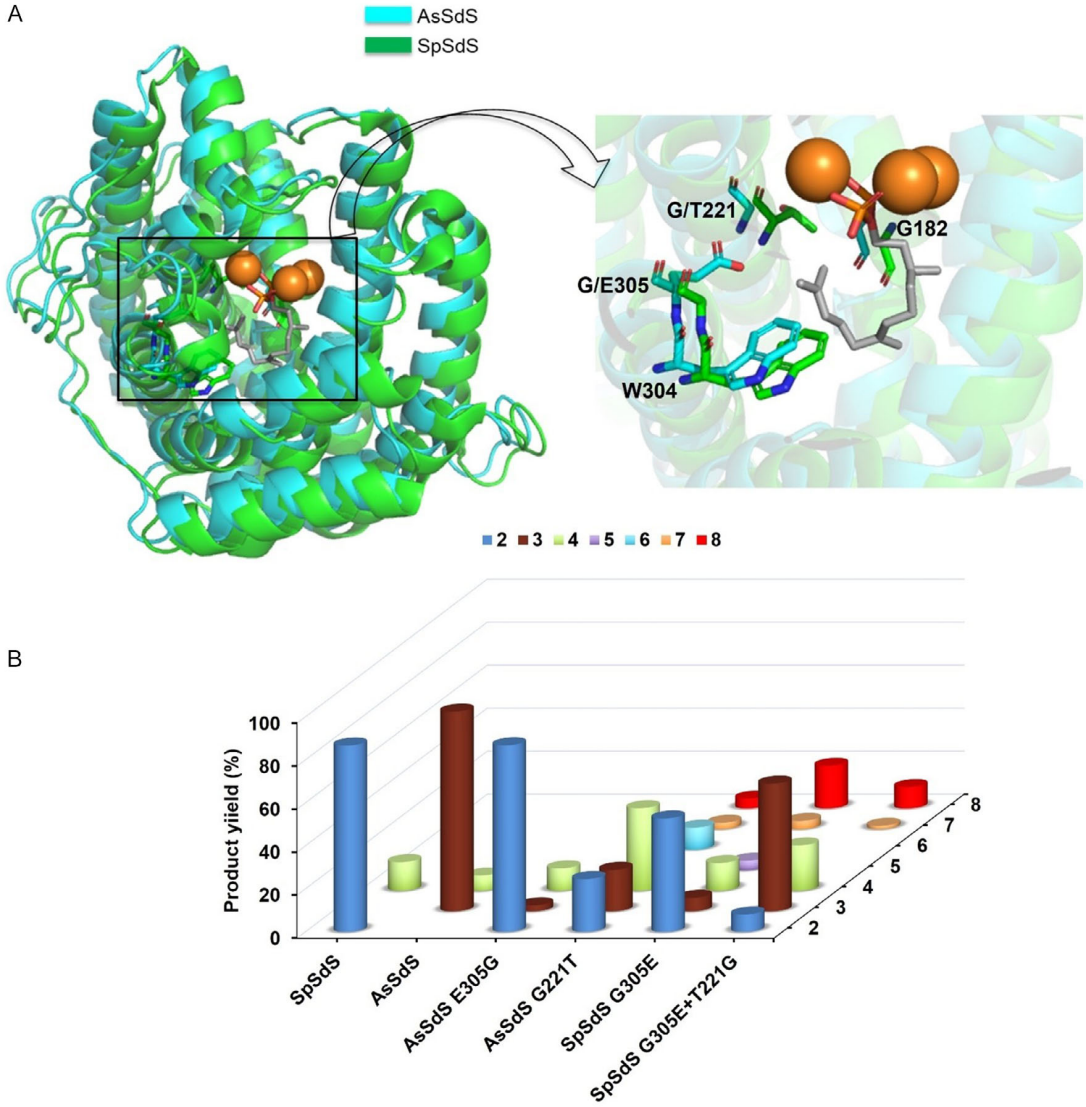
(A) Active site pocket comparison of SpSdS and AsSdS showing the notable differences in K_helix_ and H_helix_. (B) Product distribution of AsSdS, AsSdS E305G, AsSdS G221T, SpSdS, SpSdS G305E, and SpSdS G305E+T221G upon incubation with (2*E*,6*E*)‐FDP (**1**). **2**: selina‐4(15),7(11)‐diene, **3**: selina‐3,7(11)‐diene, **4**: germacrene B, **5**: δ‐selinene, **6**: uncharacterised sesquiterpene, **7**: uncharacterised sesquiterpene, **8**: selin‐7(11)‐en‐4‐ol. Gas chromatograms and mass spectra for all the variants and products are shown in Figures S3–S18.

**FIGURE 4:**
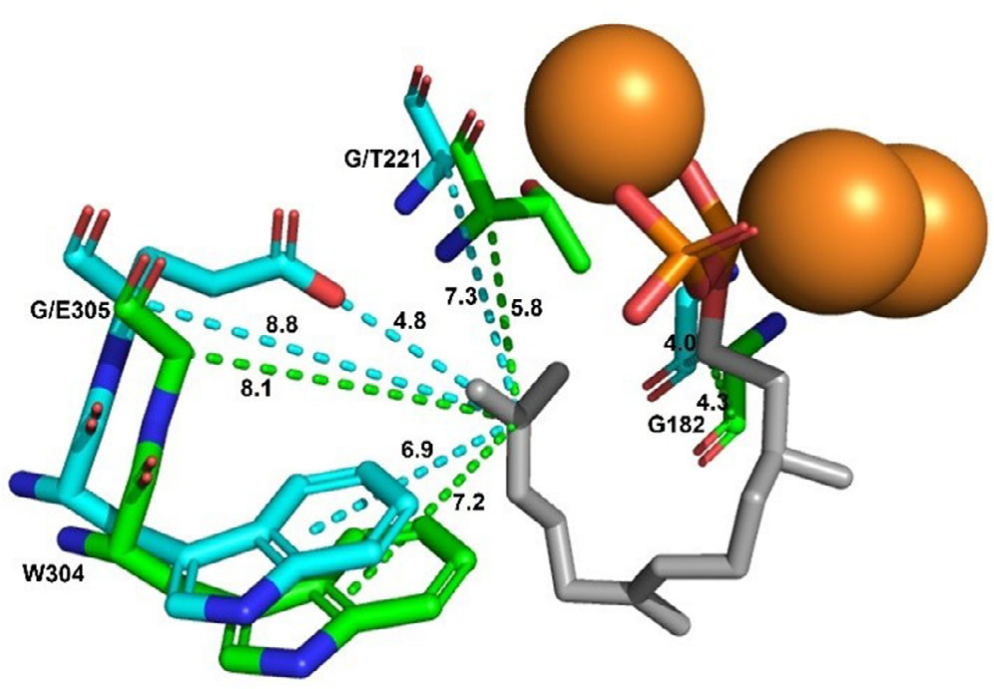
Measurement of distances (Å) from C11 (FDP) to the W304, E/G 305, G/T221 and G182 in AsSdS and SpSdS (cyan: AsSdS, green: SpSdS).

After establishing the product profile, we created a homology model of AsSdS using SpSdS (4OKZ [6], 71.7% overall sequence identity) as a template and compared their active site pockets (Figure 3A). Despite sequence variability, the active sites are very similar in both the enzymes. The most notable difference was in the K_helix_ region (305–309) (Figure 1A), with the most significant difference being the presence of E at 305 in AsSdS instead of G leading to a shift in the backbone of 0.7 Å from the C11 position (FDP numbering in Scheme 1, Figure 4). However, protrusion of E305 into the active site brings the distance to 4.8 Å between the closest oxygen atom of E305 and C11 (FDP). Another notable difference is in the H_helix_ region with the presence of glycine at 221 in AsSdS rather than T221 in SpSdS (Figure 4). These changes could affect the orientation of intermediates as well as positioning and/or availability of water molecules required to quench the final carbocation (Figure S26). To further test the role of G/E at 305 in selinadiene synthase catalysis, we created a AsSdS E305G variant. Functional characterisation of AsSdS E305G upon incubation with **1** resulted in a drastic change in the product outcome with formation of **2** (86.8%) as a major product with traces of **3** (2.8%) along with **4**, (10.4%), very similar to the SpSdS profile (**2**, 86.7% and **4**, 13.3%, Figure 3B, Table S2). Kinetic characterisation of AsSdS E305G resulted in similar kinetic parameters (*K*_M_ 1.9 ± 0.6 µM and *k*_cat_ 6.0 ± 0.6 s^−1^) to AsSdS and SpSdS (Table 1), suggesting that these changes do not impact the overall catalytic efficiency. In addition to this, the absence of linear or more relaxed products suggests that this change in not impacting the initial steps of the catalytic mechanism.

Recent work on the catalytic mechanism of SpSdS using MD simulations and isotopic labelling experiments showed that carbonyl oxygen of G182 in the kink of G_helix_ acts as a base and an acid in coordination with a trapped water molecule and helps in carbocation rearrangement as well as with deprotonation/reprotonation of the neutral intermediate germacrene B [32]. Monitoring of key interactions using QM/MM MD simulation showed that the distance between C15 and the closest oxygen of the PPi after formation of carbocation B decreased from 5.25 to 3.36 Å, allowing final deprotonation to occur from C15 to PPi, generating **2**. Functional characterisation of SpSdS and AsSdS producing different selinadienes indicates a crucial role of the residue (G/E) present at position 305 in selinadiene catalysis. This notable difference can subtly affect the helical movement, resulting in a slight change in the orientation of carbocation B (impacting the distances between PPi and C15 and C4 site of deprotonation), ultimately affecting the final deprotonation and product outcome. Furthermore, to enhance our understanding, we created a variant of AsSdS by replacing G221 with threonine (the corresponding residue in SpSdS) in H_helix_. Functional characterisation of AsSdS G221T by incubation with **1** resulted in the formation of **4** as the dominant product (38.4%) along with **8** (4.6%), **2** (24.6%) and **3** (19.3%, Figure 3B). The formation of **8** by AsSdS G221T (which also contains E at 305 naturally) is intriguing and indicates that E305 at K_helix_ and T221 at H_helix_ could both be required together to form **8**. Since SpSdS naturally contains T221 in the H_helix_, changing G305 to E led to the formation of **8**. Whereas, despite the presence of E305 naturally in AsSdS (in this G221 is naturally present) does not make **8**; instead, it forms **3** derived from the same carbocation by deprotonation at C4 (Scheme 1). Kinetic characterisation of this variant resulted in a decrease in *K*_M_ (0.5 ± 0.28 µM) and threefold reduction in *k*_cat_ (Table 1). This distinctive characteristic profile suggests that these residues could create a bridge to hold the water molecule in close vicinity of the reactive centre of carbocation B to promote water capture at C3 to form **8**.

To further test this hypothesis, we performed multiple sequence alignment of these selinadiene synthases, along with functionally characterised hydroxylating sesquiterpene synthases (Figure S2). Sequence analysis showed that all hydroxylating sesquiterpene synthases contain a polar residue at the corresponding position of G305 present in SpSdS except 7‐*epi*‐eudesmol synthase [31] and corvol ether A synthase [33]. However, the majority of them also contain a polar residue at the corresponding position of T221 in SpSdS (Figure S2), indicating that polar residues are pivotal at these positions to catalyse hydroxylation. Furthermore, we mutated T221 into glycine in SpSdS G305E variant to simulate the K_helix_ and H_helix_ of AsSdS, postulating that it would not form the required bridge and should reduce and/or stop the formation of **8** (Figure 3B). Gratifyingly, functional characterisation of this double mutant (SpSdS G305E+T221G) resulted mostly in the predicted outcome with a product profile of **3** (59.4%) as the major product with a significant reduction in **2** (8.1%). Most importantly, the level of **8** was reduced to half (9.9%) as compared to the SpSdS G305E (Figure 3B, Table S2). Kinetic characterisation of SpSdS G305E+T221G resulted in similar *K*_M_ and *k*_cat_ as compared to SpSdS (Table 1). These results clearly indicate that these two residues in their respective helices are crucial in allowing water molecule(s) to be positioned in the right place close to the reaction centre to quench the final carbocation to form **8**. However, the low yield of selin‐7(11)‐en‐4‐ol despite having E305 at K_helix_ and T221 in H_helix_ suggests that the quenching of final carbocation with water is likely dependent on multiple cooperative factors, including subtle conformational dynamics, positioning of catalytic water molecules, and interactions with other neighbouring residues.

In our previous work, we demonstrated that formation of selin‐7(11)‐en‐4‐ol (**8**) increased to become a major product when the reaction was performed under mildly acidic conditions (pH 6.0) for SpSdS G305E and that no selin‐7(11)‐en‐4‐ol formation was observed with SpSdS wild‐type. Instead, the latter produced selina‐4(15),7(11)‐diene as the sole product at lower pH and germacrene B at higher pH [20]. To test the influence of pH on the formation of **8** by AsSdS G221T, we performed a pH‐dependent product profile analysis of AsSdS and its G221T variant, following the same approach. The AsSdS profile resembled that of SpSdS, with no formation of selin‐7(11)‐en‐4‐ol. However, no significant change in the level of selin‐7(11)‐en‐4‐ol was observed in the case of AsSdS G221T. At higher pH, germacrene B became the predominant product (Figure S11). These results further confirm that selin‐7(11)‐en‐4‐ol formation is not solely dependent upon pH change and support the hypothesis that there is a requirement for polar residues at these two respective helices, which are essential for guiding the water molecules to quench the final carbocation.

In conclusion, we identified a selinadiene synthase through multiple sequence alignment, which showed a notable difference at position 305, containing glutamate instead of glycine and glycine instead of threonine at position 221. Functional characterisation of this enzyme resulted in a new sesquiterpene synthase producing selina‐3,7(11)‐diene as a major product. Through mutagenesis experiments, we have demonstrated the critical role of the K_helix_ residue G/E305 in the catalytic mechanism for selinadienes, guiding the final deprotonation and/or water capture in two different selinadiene synthases, resulting in a different product but only effecting deprotonation or water attack without skeletal alterations of the product. Further, we show that replacing G221 with T in the H_helix_ in conjunction with E305 in AsSdS led to the formation of selin‐7(11)‐en‐4‐ol through water capture previously observed as a product generated by the SpSdS G305E variant. However, replacing the corresponding residues in SpSdS (G305E and T221G) significantly reduces selin‐7(11)‐en‐4‐ol formation, instead giving selina‐3,7(11)‐diene as a major product derived, by deprotonation, from the same carbocation B. These findings suggest that E305 at K_helix_ plays a crucial role in determining the final deprotonation from carbocation B in selinadiene synthases. However, T221 at H_helix_ together with E305 are crucial for guiding the water molecule in the close vicinity of the reaction centre to quench the reactive carbocation and form a hydroxylated product (**8**) in selinadiene synthase catalysis. This work exemplifies how subtle yet increasingly predictable changes within the active‐site pocket can drive functional switches, generating distinct product profiles. This work represents significant progress towards predictable engineering of terpene synthases to manipulate water capture and hence final product outcomes and will have broad applications in sustainable biocatalytic production of this class of complex bioactive molecules.

## Supporting Information

Additional supporting information can be found online in the supporting information section. **Supporting Fig. S1A**: Multiple sequence alignment of representative selinadiene synthases (182 sequences) with amino acid sequence identity ≥ 70% using MEGA11. **Supporting Fig. S1B**: Amino acid sequence alignment of selina‐4(15),7(11)‐diene synthases (SpSdS: B5HDJ6) and selina‐3,7(11)‐diene synthase (AsSdS: WP_127359384) showing overall sequence identity. Region targeted for study in this manuscript at K_helix_ (G/E305) and H_helix_ (G/T221) are marked with arrows in red colour. **Supporting Fig. S2**: Multiple sequence alignment of functionally characterised hydroxylating sesquiterpene synthases along with selinadiene synthases using MEGA11. **Supporting Fig. S3**: TIC of pentane extractable products arising from the incubation of SpSdS with (2*E*,6*E*)‐FDP (**1**), producing selina‐4(15),7(11)‐diene (**2**) as the major product along with germacrene B (**4**) as a minor product. **Supporting Fig. S4**: TIC of pentane extractable product arising from the incubation of AsSdS with (2*E*,6*E*)‐FDP (**1**), producing selina‐3,7(11)‐diene (**3**) as the major product along with minor quantities of germacrene B (**4**). **Supporting Fig. S5**: TIC of pentane extractable product arising from the incubation of AsSdS E305G with (2*E*,6*E*)‐FDP (**1**), producing selina‐4(15),7(11)‐diene (**2**) as the major product along with minor quantities of selina‐3,7(11)‐diene (**3**) and germacrene B. **A)** Full chromatogram. **B)** Zoomed chromatogram for improved clarity. **Supporting Fig. S6**: TIC of pentane extractable product arising from the incubation of AsSdS G221T with (2*E*,6*E*)‐FDP (**1**), producing germacrene B (**4**) as a major product with reduced level of selina‐3,7(11)‐diene (**3**) along with the formation of selina‐4(15),7(11)‐diene (**2**) and minor quantities of selin‐7(11)‐en‐4‐ol (**8**). **A)** Full chromatogram. **B)** Zoomed chromatogram for improved clarity. **Suporting Fig. S7**: TIC of pentane extractable product arising from the incubation of SpSdS G305E^[3]^ with (2*E*,6*E*)‐FDP (**1**), producing selina‐4(15),7(11)‐diene (**2**) as a major product along with hydroxylated sesquiterpene selin‐7(11)‐en‐4‐ol (**8**) and traces of selina‐3,7(11)‐diene (**3**), germacrene B (**4**) and δ‐selinene (**5**). **Supporting Fig. S8**: TIC of pentane extractable product arising from the incubation SpSdS G305E + T221G with (2*E*,6*E*)‐FDP (**1**), producing selina‐3,7(11)‐diene (**3**) as a major product along with minor quantities of selina‐4(15),7(11)‐diene (**2**) and reduced level of selin‐7(11)‐en‐4‐ol (**8**). **A)** Full chromatogram. **B)** Zoomed chromatogram for improved clarity. **Supporting Fig. S9**: Preparative scale incubation of AsSdS for NMR spectroscopic characterisation. TIC of pentane extractable product arising from incubation of AsSdS with (2*E*,6*E*)‐FDP (**1**). The major sesquiterpene fraction was confirmed as selina‐3,7(11)‐diene (**3**) by NMR spectroscopy and comparing the observed spectral with data reported in the literature [9–11]. **Supporting Fig. S10**: Co‐injection of AsSdS and SpSdS assay samples. **A)** TIC of pentane extractable product arising from incubation of SpSdS with (2*E*,6*E*)‐FDP (**1**) producing selina‐4(15),7(11)‐diene (**2**) as a major product along with germacrene B (**4**) as a minor product. **B)** TIC of pentane extractable product arising from incubation of AsSdS with (2*E*,6*E*)‐FDP (**1**) producing selina‐3,7(11)‐diene (**3**) as the major product along with minor quantities of germacrene B (**4**). **C)** TIC of co‐injection of pentane extractable product arising from the incubation of SpSdS and AsSdS (2*E*,6*E*)‐FDP (**1**). **Supporting Fig. S11**: pH studies of AsSdS wild‐type and AsSdS G221T. **A)** TICs of pentane extractable product arising from incubation of AsSdS with (2*E*,6*E*)‐FDP (**1**) producing selina‐3,7(11)‐diene (**3**) as a major product at lower pH and germacrene B (**4**) as a minor product at higher pH. **B)** TICs of pentane extractable product arising from incubation of AsSdS G221T with (2*E*,6*E*)‐FDP (**1**) producing selinadienes: selina‐4(15),7(11)‐diene (**2**), selina‐3,7(11)‐diene (**3**) at lower pH with small percentage of selin‐7(11)‐en‐4‐ol (**8**) and germacrene B (**4**) as major product at higher pH. **Supporting Fig. S12**: EI^+^ Mass spectrum of the compound eluting at 13.06 min in the gas‐chromatogram (selina‐4(15),7(11)‐diene, **2**) from the incubation of (2*E*,6*E*)‐FDP (**1**) with SpSdS and AsSdS E305G. **Supporting Fig. S13**: EI^+^ Mass spectrum of the compound eluting at 13.20 min in the gas‐chromatogram (selina‐3,7(11)‐diene, **3**) from the incubation of (2*E*,6*E*)‐FDP (**1**) with AsSdS, AsSdS G221T, AsSdS E305G, SpSdS G305E and SpSdS G305E + T221G. **Supporting Fig. S14**: EI^+^ Mass spectrum of the compound eluting at 13.38 min in the gas‐chromatogram (germacrene B, **4**) from the incubation of (2*E*,6*E*)‐FDP (**1**) with SpSdS, AsSdS and other variants. **Supporting Fig. S15**: EI^+^ Mass spectrum of the compound eluting at 12.37 min in the gas‐chromatogram (δ‐selinene, **5**) from the incubation of (2*E*,6*E*)‐FDP (**1**) with SpSdS G305E. **Supporting Fig. S16**: EI^+^ Mass spectrum of the compound eluting at 12.73 min in the gas‐chromatogram (uncharacterised sesquiterpene, **6**) from the incubation of (2*E*,6*E*)‐FDP (**1**) with AsSdS G221T. **Supporting Fig. S17**: EI^+^ Mass spectrum of the compound eluting at 12.88 min in the gas‐chromatogram (uncharacterised sesquiterpene, **7**) from the incubation of (2*E*,6*E*)‐FDP (**1**) with SpSdS G305E and AsSdS G221T. **Supporting Fig. S18**: EI^+^ Mass spectrum of the compound eluting at 15.17 min in the gas‐chromatogram (selin‐7(11)‐en‐4‐ol, **8**) from the incubation of (2*E*,6*E*)‐FDP (**1**) with SpSdS G305E, AsSdS G221T and SpSdS G305E+T221G. **Supporting Fig. S19**: Representative Michaelis–Menten plot for the conversion of [1‐^3^H]‐FDP by AsSdS. **Supporting Fig. S20**: Representative Michaelis–Menten plot for the conversion of [1‐^3^H]‐FDP by AsSdS E305G. **Supporting Fig. S21**: Representative Michaelis–Menten plot for the conversion of [1‐^3^H]‐FDP by AsSdS G221T. **Supporting Fig. S22**: Representative Michaelis–Menten plot for the conversion of [1‐^3^H]‐FDP by SpSdS G305E +T221G. **Supporting Fig. S23A**: ^1^H NMR spectrum (500 MHz, CDCl_3_, 298K) of selina‐3,7(11)‐diene (**3**) full. **Supporting Fig. S23B**: ^1^H NMR spectrum (500 MHz, CDCl_3_, 298K) of selina‐3,7(11)‐diene (**3**) expansions. **Supporting Fig. S24A**: ^13^C NMR spectrum (125 MHz, CDCl_3_, 298K) of selina‐3,7(11)‐diene (**3**). Peak at 29.71 represents the contamination of grease. **Supporting Fig. S24B**: Expansion of the ^13^C NMR spectrum (125 MHz, CDCl_3_, 298K) of selina‐3,7(11)‐diene (**3**). Peak at δ_C_ = 29.71 represents a small contamination of grease. **Supporting Fig. S25**: DEPT 135 NMR spectrum (125 MHz, CDCl_3_, 298K) of selina‐3,7(11)‐diene (**3**). Peak at δ_C_ = 29.71 represent a small contamination of grease. **Supporting Fig. S26**: Measurement of distances (Å) from E/G 305 at K_helix_ and G/T221 in H_helix_ in AsSdS and SpSdS (cyan: AsSdS, green: SpSdS) with trapped water molecules indicating that could create a bridge required for quenching the final carbocation to form selin‐7(11)‐en‐4‐ol (**8**) by selinadiene synthase variants containing E305 at K_helix_ and T221 at H_heli__x_. Trapped water molecules are highted as stick in red colour. AsSdS homology model was overlaid with crystal structure reported for SpSdS (pdb: 4OKZ). **Supporting Fig. S27**: SDS‐PAGE analysis of purification of AsSdS using Ni‐NTA column chromatography. **Supporting Fig. S28**: SDS‐PAGE analysis of purification of AsSdS E305G using Ni‐NTA column chromatography. **Supporting Fig. S29**: SDS‐PAGE analysis of purification of AsSdS wild‐type and AsSdS variant G221T using Ni‐NTA column chromatography. **Supporting Table S1**: Primer sequences for mutagenesis of AsSdS and SpSdS. Nucleotide sequences changed are marked bold and underlined. SpSdS G305E+T221G variant was generated by mutating the SpSdS G305E variant as template using the primers for incorporating T221G mutation. **Supporting Table S2**: Product ratios table for AsSdS, SpSdS and variants upon incubation with (2*E*,6*E*)‐FDP (**1**), **2**: selina‐4(15),7(11)‐diene, **3**: selina‐3,7(11) diene, **4**: germacrene B, **5**: δ‐selinene, **6**: uncharacterised sesquiterpene, **7**: uncharacterised sesquiterpene, **8**: selin‐7(11)‐en‐4‐ol**.**

## Funding

This study was supported by Biotechnology and Biological Sciences Research Council (BB/R001596/1 and BB/R019681/1) and Cardiff University.

## Conflicts of Interest

The authors declare no conflicts of interest.

## Supporting information

Supplementary Material

## Data Availability

The data that support the findings of this study are openly available in Cardiff University Research Data Repository at https://doi.org/10.17035/cardiff.29136794.
