# Supplementary material for: Designed Water Capture in Terpene Synthase Catalysis
Source: Chembiochem. 2026 Mar 12;27(5):e70265. doi: 10.1002/cbic.70265 (PMC12980470; doi:10.1002/cbic.70265)
Supplement: Supplementary file 1 — Supplementary Material [file CBIC-27-e70265-s001.pdf]

Supporting Information

Designed water capture in terpene synthase catalysis

Prabhakar L. Srivastava,[a] David J. Miller,[a] and Rudolf K. Allemann*[a]

[a]School of Chemistry, Cardiff University, Main Building, Park Place, Cardiff CF10 3AT, United Kingdom

*Correspondences to: allemannrk@cardiff.ac.uk

Table of Contents

1.	General materials and methods	S2
2.	Multiple sequence alignment and construction of phylogenetic tree	S3
3.	Homology modelling	S3
4.	Construction of AsSdS variants	S4
5.	Protein expression and purification of AsSdS and variants	S4
6.	Enzymatic incubations and product analysis	S6
7.	Kinetic characterisation of AsSdS and variants	S6
8.	Preparative scale incubations and product characterisation	S7
9.	Tables	S8
10.	Multiple sequence alignment	S9
11.	Total ion chromatograms (TICs)	S19
12.	GCMS Mass Spectra	S28
13.	Kinetic data	S32
14.	NMR spectra	S33
15.	Computational image	S37
16.	SDS-PAGE analysis	S38
17.	References	S40

1. General materials and methods

The full-length gene sequence (1-380 aa) of WP_127359384 annotated as selina-4(15),7(11)-diene synthase (in non-redundant database) from *Actinacidiphila soli*[1] was codon optimised for bacterial expression in *Escherichia coli* and synthesised in a pET28a vector frame between EcoRI and HindIII restriction site from Genscript with an N-terminal 6x His tag for affinity purification.

An unstained protein molecular weight marker (14.4-116.0 kDa) was used as an indicative standard to identify the molecular weight of proteins by 12% SDS-PAGE. PrimeSTAR® master mix was purchased from TaKaRa for PCR amplification and mutagenesis. Primers for mutagenesis were purchased from Sigma Aldrich (UK). Incorporation of mutations at the specific positions was confirmed by DNA sequence analysis with sequencing results from Eurofins. [1-^{3}H]-FDP (20 Ci/mmol) was purchased from American Radiolabelled chemicals, Inc. Commercial [1-^{3}H]-FDP was diluted by adding unlabelled (2*E*,6*E*)-FDP to give a final specific activity of 24000 dpm/µM.

^{1}H, ^{13}C and DEPT 135 NMR spectra were measured on a Bruker Ultrashield 500 NMR spectrometer and are reported as chemical shifts in parts per million downfield from tetramethylsilane.

(2*E*,6*E*)-FDP was chemically synthesised in our laboratory as described previously[2] for enzymatic incubations and product ratio analysis.

Gas chromatography coupled with mass spectrum (GC-MS) was performed on a Perkin Elmer Clarus 680 GC fitted with a Perkin Elmer Elite-1 column 100% dimethyl polysiloxane (30 m x 0.25 mm internal diameter) and a Perkin Elmer Clarus SQ 8 C mass spectrometer: The elution program used an injection port

temperature of 100 °C; split ratio of 19:1; initial column temperature at 80 °C hold 2 min, then ramp of 8 °C/min to 280 °C and final hold for 3 min with a flow rate of 1 mL He/min.

2. Multiple sequence alignment and construction of phylogenetic tree

In order to understand the role of glutamate at position 305 of SpSdS G305E variant which resulted in the formation of predefined sesquiterpene alcohol selin,3-7(11)-en-4-ol,[3] we performed a BLAST search against non-redundant (nr) database using selina-4(15),7(11)-diene synthase (B5HDJ6) to search for any representative sequences containing different residues at the corresponding position of G305. A total of 182 protein sequences were selected showing at least 70% identity at amino acid level and multiple sequence alignment was carried out using CLUSTALW. The aligned sequences were used to construct a neighbor-joining phylogenetic tree by MEGA11 program using default parameters.[4]

3. Homology modelling

A homology model for selina-3,7(11)-diene synthase (AsSdS) was constructed by SWISS-MODEL workspace,[5] using the crystal structure of selina-4(15),7(11)-diene synthase (SpSdS, 4OKZ)[6] in complex with 2,3-dihydrofarnesyl diphosphate as a template with default parameters. This homology model of AsSdS was overlapped with the crystal structure of SpSdS and their overall structure and active site pockets were compared and visualised using PyMOL Molecular Graphics System, Schrodinger.

4. Construction of AsSdS variants

Overhang mutation primers were designed for the selected amino acid present in the active site pocket (**Table S1**). For PCR amplification the following mixture was used: 12.5 µL PrimeSTAR® master mix, 0.5 µL of each forward and reverse primer (10 µM stock), 1 µL template DNA (50 ng plasmid), 1 µL DMSO and 10 µL water to make up the final volume up to 25 µL. PCR amplification parameter: initial denaturation for 5 min at 95 °C, followed by 32 PCR cycle using initial denaturation for 30 sec at 95 °C, annealing for 20 sec at 60-65 °C, extension at 70 °C for 7 min. Final extension was performed for 15 min at 72 °C. After completion of the PCR, 1 µL of DpnI (NEB) was added to each reaction mixture and further incubated at 37 °C for 4 h. Aliquots of 10 µL of samples were transformed into XL1 blue chemical competent cells and plated onto Luria Agar (LA) plates containing kanamycin (50 µg/mL) and incubated at 37 °C for overnight to select the positive clones. Single colonies were inoculated in Luria Broth (LB) medium containing kanamycin (50 µg/mL) and incubated at 37 °C overnight. Plasmids were isolated using a plasmid miniprep kit (Qiagen). The variant sequences were verified via sequencing using the forward (T7 promoter) or the reverse primer (T7 terminator).

5. Protein expression and purification of AsSdS, SpSdS and their variants

pET28a harbouring the genes encoding selina-3,7(11)-diene synthase from *Actinacidiphila soli* (AsSdS), selina-4(15),7(11)-diene synthase from *Streptomyces prestinaspiralis* (SpSdS)[6] and their variants were introduced in chemically competent BL21(DE3) cells for heterologous protein expression and positive clones were selected on LA plates containing kanamycin (50 µg/mL).

A single colony was used to inoculated 20 mL of LB media containing 50 µg/mL of kanamycin and incubated overnight at 37 °C and 200 rpm. The overnight grown culture was then transferred to 500 mL of terrific broth (TB) media containing 50 µg/mL of kanamycin and incubated at 37 °C and 200 rpm until the optical density (OD) at 600 nm reached 1.0-1.2. At this stage, cultures were induced by addition of isopropyl β-D-1-thiogalactopyranoside (IPTG, final concentration 0.2 mM) and incubated overnight at 16 °C and 200 rpm for protein expression. The cells were harvested by centrifugation at 5000*g* for 20 min at 20 °C and pellets were stored in -20 °C until further use. The cell pellets were resuspended in 30 mL lysis buffer /g of cell mass (20 mM Tris, pH 8.0, 500 mM NaCl, 10% glycerol, 1 mg/mL lysozyme and 1 mM PMSF) and incubated on ice for 30 min. The cell suspensions were further subjected to sonication (amplitude 40%, pulse on 5 s and pulse off 10 s for 5 min). Sonicated samples were centrifuged at 18000*g* for 45 min at 4 °C to remove the cell debris. The supernatant solutions were filtered through 0.2 µm syringe filters and applied to a preequilibrated Ni-NTA affinity drip column (QIAGEN, 5 mL) with lysis buffer. The column was washed by applying a wash buffer containing gradient of imidazole (5 column volumes of each 0, 20, 40 mM in lysis buffer) to remove non-specific protein contaminants. Proteins were eluted with 5 column volumes of 250 mM imidazole solution in lysis buffer. All fractions were analysed by SDS-PAGE to check the protein purity. Eluted proteins were desalted to remove imidazole on a Hiprep 26/10 desalting column against the 20 mM Tris, pH 8.0, 100 mM NaCl, glycerol 10% buffer using a FPLC (ACTA). The Bradford protein assay[7] was used to determine the concentration of desalted protein using commercial bovine serum albumin as the calibration standard.

6. Enzymatic incubations and product analysis

Enzymatic assays for the functional characterisation of AsSdS, SpSdS and their variants were performed using 5 µM of purified protein in 50 mM Tris buffer, pH 8.0, containing 5 mM $MgCl_2$, 5.0 mM 2-mercaptoethanol (βME) and 100 µM (2*E*,6*E*)-FDP on a 500 µL scale and overlaid with 1 mL of n-pentane. All the reaction mixtures were incubated overnight at room temperature with gentle agitation. After the incubation, assay mixtures were vortexed to extract the products and the n-pentane layer was transferred to fresh vial. The pentane extracts were analyzed by GC-MS using the method described above. The products formed were verified by NIST Library Mass Spectra matches and NMR spectroscopic analysis. All the enzymatic incubations were performed in three biological replicates. Chromatogram peak areas from each replicate for all the products generated by each variant were used to calculate the product ratios of each variant (**Table S2**).

7. Kinetic characterisation of AsdS, SpSdS and variants

Steady-state kinetics assays for AsSdS, SpSdS and their variants were carried out using [1-^{3}H]-(2*E*,6*E*)-FDP (240000 dpm nmol^{-1}) in Tris buffer pH 8.0 in a similar way as previously described.[8] Briefly, reactions (250 µL) were initiated by addition of purified enzyme (100 nM, final concentration) to assay buffer solutions containing [1-^{3}H]-(*E*,*E*)-FDP (0.02-20 µM) at 0 °C and overlaid with 1 mL of hexane. The resulting reaction mixture were incubated at 30 °C for 10 minutes with shaking. After incubation, reaction mixtures were transferred on ice and immediately quenched by addition of EDTA (50 µL, 0.5 M) and vortexing for 30 s. The organic layer was then passed through a small silica column (~500 mg) into 15 mL of EcoScintTM fluid (National Diagnostics). The aqueous

portion was further extracted two times with 1 mL of hexane/diethyl ether (11:1) by vortexing for 10 s and the organic layers were passed through the same silica column into the 15 mL of EcoScintTM fluid. At the end, the silica column was washed with 1 mL of 11:1 hexane/diethyl ether. The combined organic extracts in EcoScintTM fluid was analyzed on a scintillation counter (Packard 2500 TR^{TM}) in ^{3}H mode for 4 min per sample. The kinetic constants (K_M and k_{cat}) for each variant were calculated by fitting the data obtained from three technical replicates to the Michaelis-Menten equation, using Systat Sigmaplot.

8. Preparative scale incubations and product characterisation

For the characterisation of major sesquiterpene produced from AsSdS, preparative scale incubations were carried out using 10 µM of purified protein with 0.35 mM FDP in Tris-HCl buffer, pH 8.0, 5 mM $MgCl_2$ and 5 mM 2-mercaptoethanol (βME) in a total reaction volume of 100 mL. This solution was overlaid with 100 mL of n-pentane and incubated at room temperature for 24 h with gentle stirring. Reactions were performed in duplicates. After incubation, all the reaction mixtures were pooled, extracted twice with 100mL n-pentane each time, and the n-pentane solution was concentrated and analysed by GC-MS. The resulting solution was filtered, and solvent removed carefully under reduced pressure (300 mbar, 30 °C water bath) to give 6.5 mg (46%) of colourless oil. For NMR spectroscopic analysis the sample was dissolved in $CDCl_3$, and NMR spectrosopic data were recorded for ^{1}H, ^{13}C and DEPT135 using a Bruker Ultrashield 500 NMR spectrometer. Selina-3,7(11)-diene (**3**) was characterised by comparing our data with the reported NMR spectra in the literature.[9–11]

9. Tables

Table S1: Primer sequences for mutagenesis of AsSdS and SpSdS. Nucleotide sequences changed are marked bold and underlined. SpSdS G305E+T221G variant was generated by mutating the SpSdS G305E variant as template using the primers for incorporating T221G mutation.

S. No	Name	Forward Primer	Reverse Primer
**1**	**AsSdS E305G**	ATTGG**GGG**ATCACCAGCATTCGTTATACC	GGTGAT**CCC**CCAATCCTGCGCACCACG
**2**	**AsSdS G221T**	ATCATT**ACT**TGGGACAACGATATCCTGAGCC	GTCCCA**AGT**AATGATGAAGTGCGCCATTTCACC
**3**	**SpSdS T221G**	TCATT**GGC**TGGGACAACGATATCTTTAGCTATC	TCCCA**GCC**AATGATGAAGCTCGCCATCTCCGCAACC

Table S2: Product ratios table for AsSdS, SpSdS and variants upon incubation with (2*E*,6*E*)-FDP (**1**), **2**: selina-4(15),7(11)-diene, **3**: selina-3,7(11) diene, **4**: germacrene B, **5**: δ-selinene, **6**: uncharacterised sesquiterpene, **7**: uncharacterised sesquiterpene, **8**: selin-7(11)-en-4-ol.

**Retention time**	**13.06**	**13.2**	**13.38**	**12.37**	**12.63**	**12.88**	**15.17**
	**2**	**3**	**4**	**5**	**6**	**7**	**8**
**SpSdS**	86.7	13.3	--	--	--	--	--
**AsSdS**	--	92.9	7.1	--	--	--	--
**AsSdS E305G**	86.8	2.8	10.4	--	--	--	--
**AsSdS G221T**	24.6	19.3	38.4	--	10.2	2.9	4.6
**SpSdS G305E**[3]	52.9	6.3	12.9	4.4	--	3.8	19.8
**SpSdS G305E+T221G**	8.1	59.4	21.1	--	--	1.5	9.9

10. Multiple sequence alignment

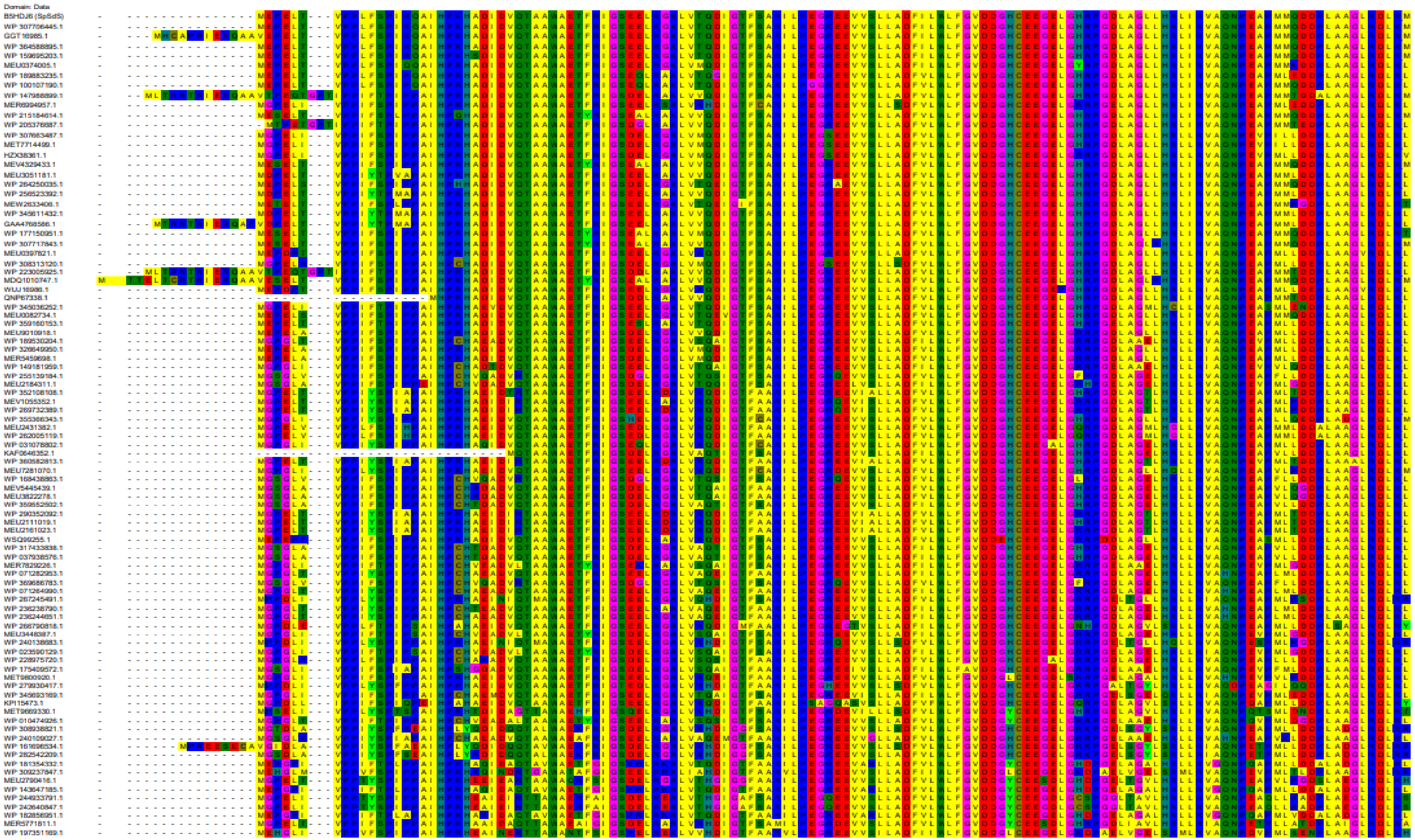

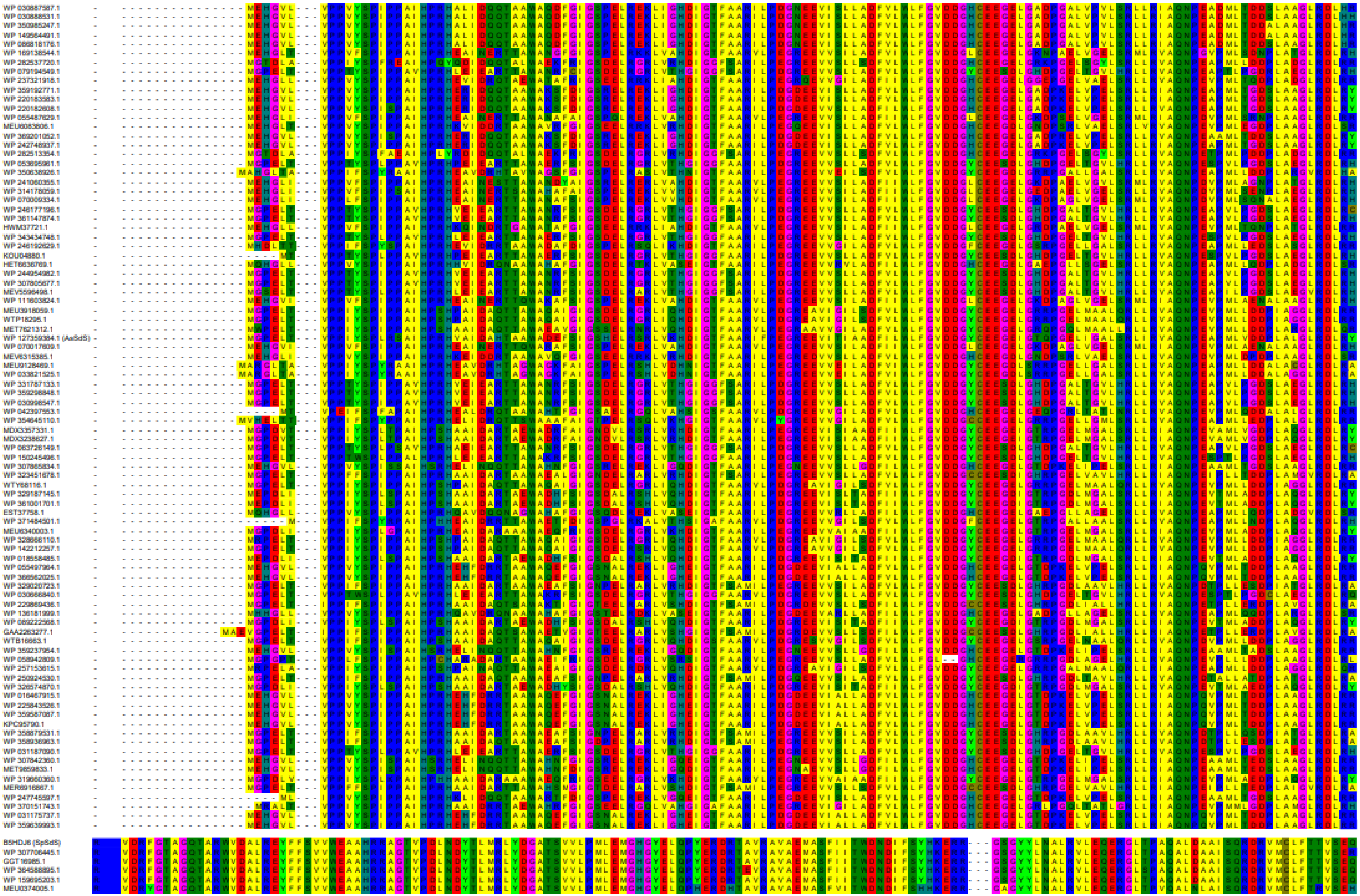

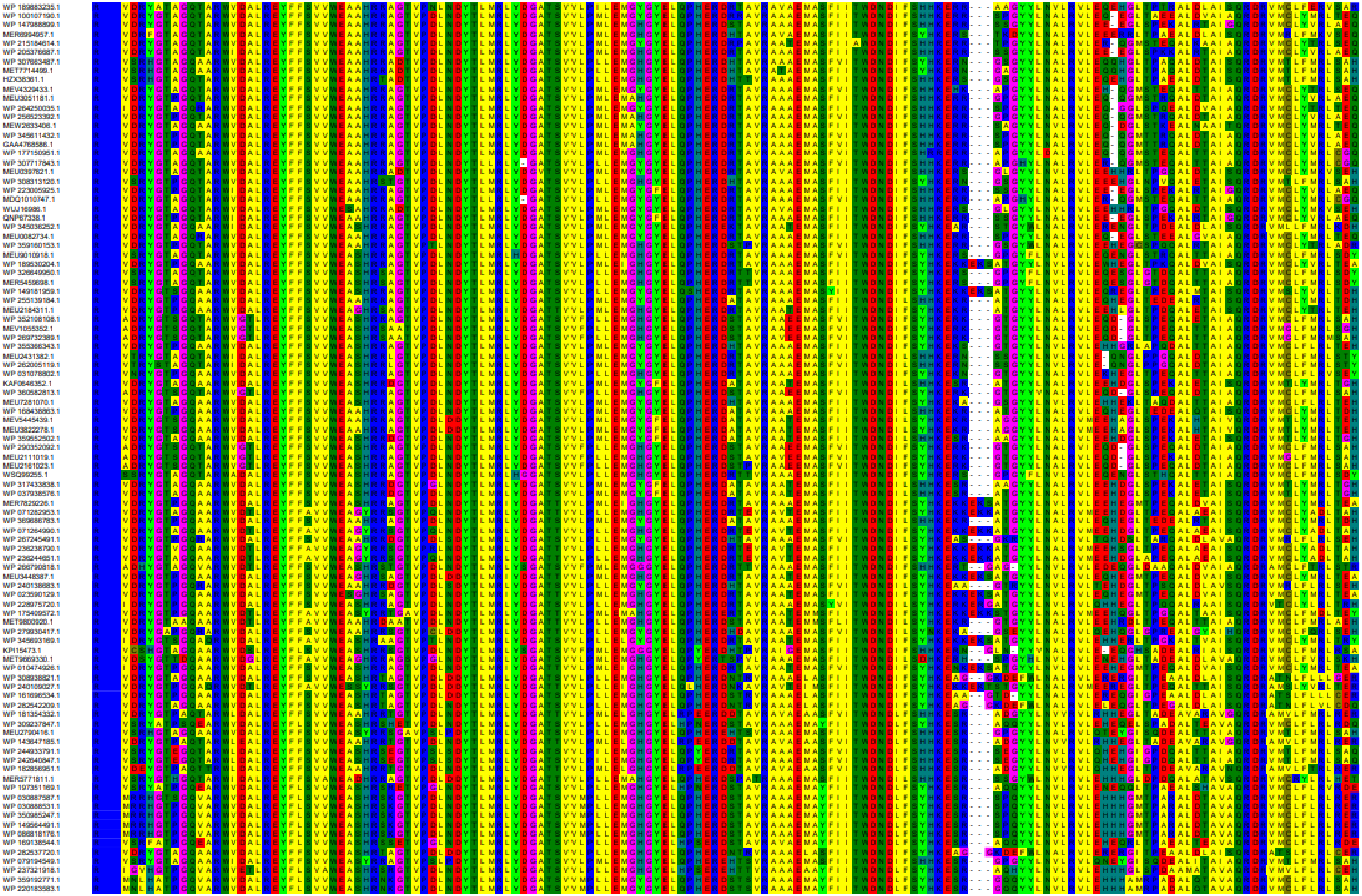

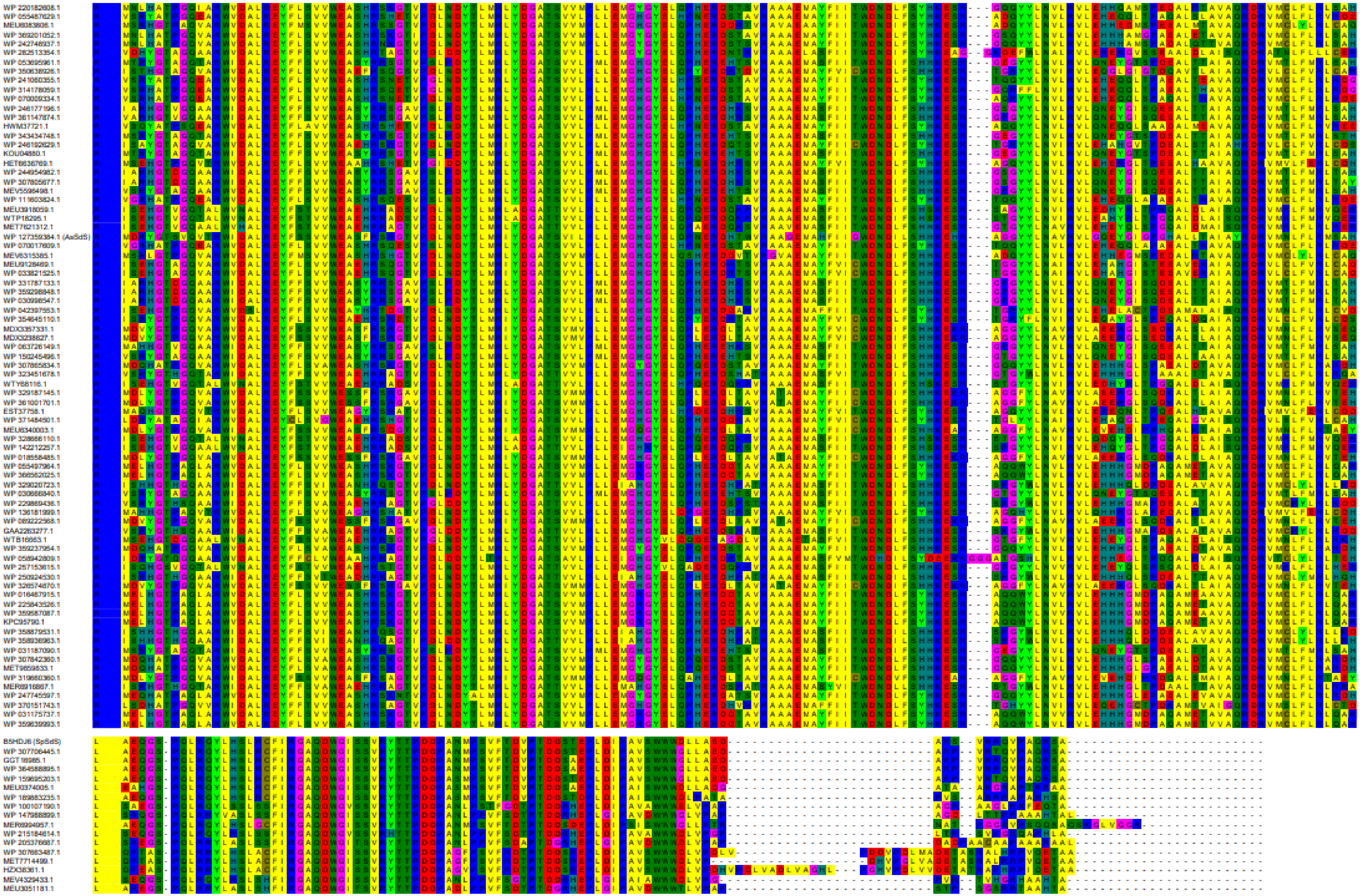

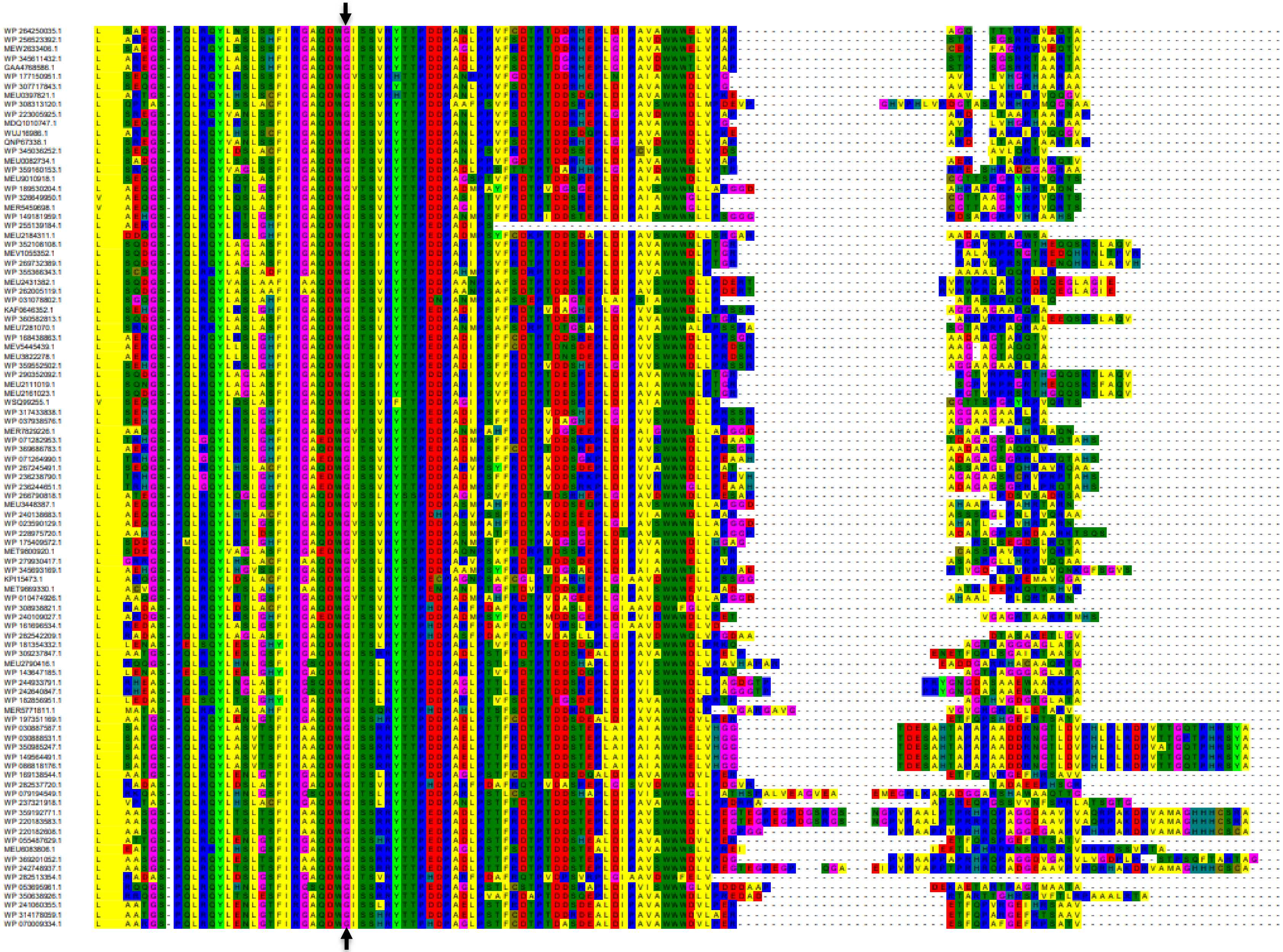

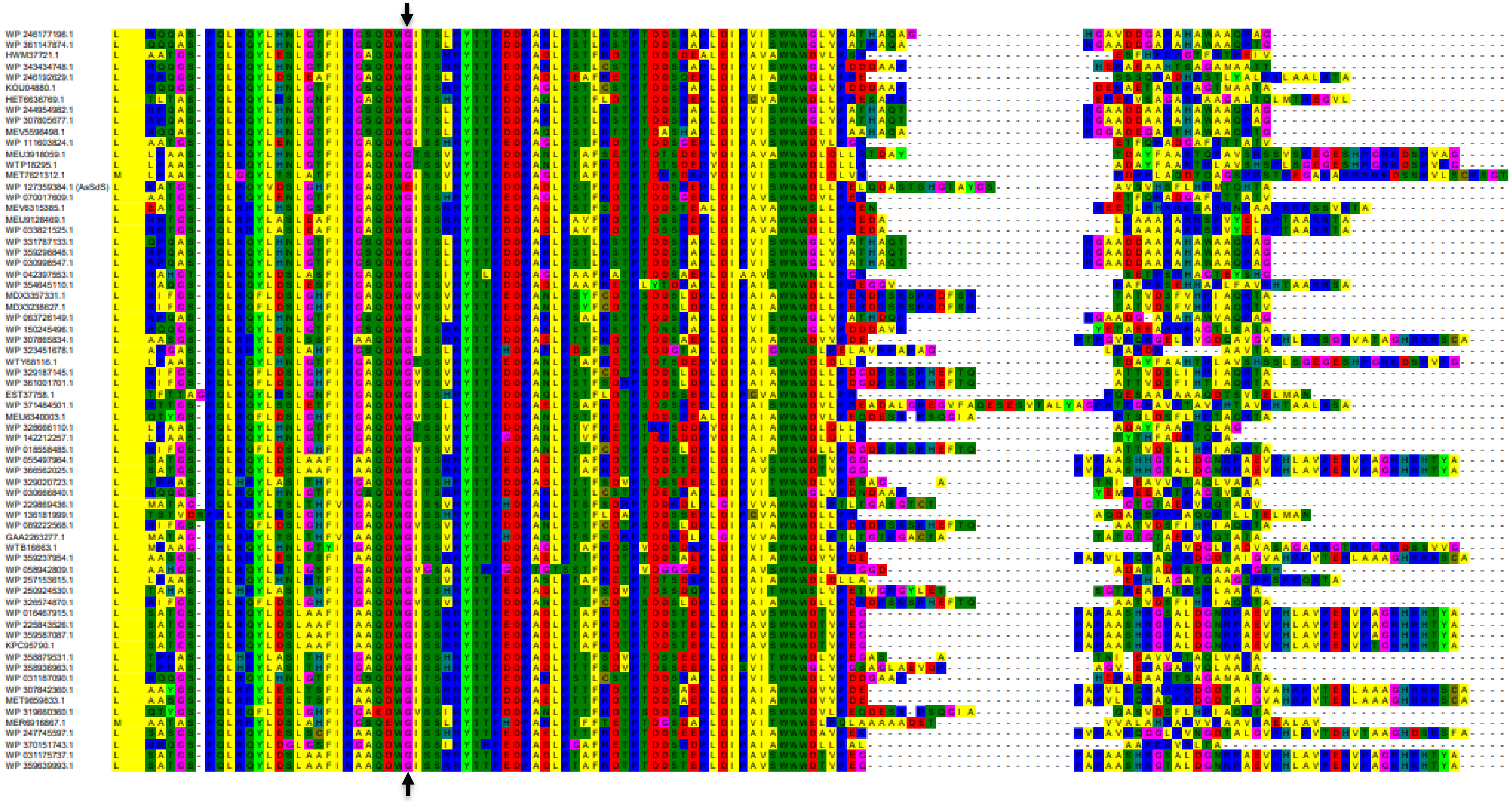

Figure S1A: Multiple sequence alignment of representative selinadiene synthases (182 sequences) with amino acid sequence identity ≥ 70% using MEGA11.

```
B5HDJ6              1 MEPELTVPPLFSPIRQAIHPKHADIDVQTAAWAETFRIGSEELRGKLVTQ     50
                      |.|||||||::||:|.||||:|..||..|||||:.|.|||.|||.:||..
WP_127359384.       1 MGPELTVPPIYSPLRSAIHPRHVAIDAHTAAWADEFSIGSHELRSRLVKH     50

B5HDJ6             51 DIGTFSARILPEGREEVVSLLADFILWLFGVDDGHCEEGELGHRPGDLAG    100
                      |||||:|||.|||||||:::.|||||||||||||:|||||:|.:||:|.|
WP_127359384.      51 DIGTFAARIFPEGREEVITIAADFILWLFGVDDGYCEEGEIGTQPGELIG    100

B5HDJ6            101 LLHRLIRVAQNPEAPMMQDDPLAAGLRDLRMRVDRFGTAGQTARWVDALR    150
                      .|.|||||||||||||:.|||||.||||||.|:||:||:.|..||:||||
WP_127359384.     101 ALSRLIRVAQNPEAPMLVDDPLALGLRDLRYRMDRYGTSVQVTRWIDALR    150

B5HDJ6            151 EYFFSVVWEAAHRRAGTVPDLNDYTLMRLYDGATSVVLPMLEMGHGYELQ    200
                      |||.||||||:.|..|||||||||||||:|||||||:||:||||||||||
WP_127359384.     151 EYFSSVVWEASFRSRGTVPDLNDYTLMRIYDGATSVILPLLEMGHGYELQ    200

B5HDJ6            201 PYERDRTAVRAVAEMASFIITWDNDIFSYHKERRGSGYYLNALRVLEQER    250
                      |.|||.|.|||..|||.|||.|||||.|:|||.|..||||||:|||.||.
WP_127359384.     201 PNERDHTWVRAAGEMAHFIIGWDNDILSHHKEHRAGGYYLNAVRVLGQEY    250

B5HDJ6            251 GLTPAQALDAAISQRDRVMCLFTTVSEQLAEQGSPQLRQYLHSLRCFIRG    300
                      |:.|..||..||:.|||||.||..:|..|...||||||||:.||..||||
WP_127359384.     251 GIGPGHALLTAIAYRDRVMNLFLRMSAHLKATGSPQLRQYVDSLGHFIRG    300

B5HDJ6            301 AQDWGISSVRYTTPDDPANMPSVFTDVPTDDSTEPLDIPAVSWWWDLLAE    350
                      ||||.|:|:|||||||||::||.|.|.|||||.||||||.:|||||||.|
WP_127359384.     301 AQDWEITSIRYTTPDDPADLPSTFRDTPTDDSPEPLDIPVISWWWDLLPE    350

B5HDJ6            351 DARSVRRQVPAQRSA---------------    365
                      ...:......|..||
WP_127359384.     351 LQDASTSHGTAYGSAVSVHSFLHPMTQHTA    380
```

Figure S1B: Amino acid sequence alignment of selina-4(15),7(11)-diene synthases (SpSdS: B5HDJ6) and selina-3,7(11)-diene synthase (AsSdS: WP_127359384) showing overall sequence identity. Region targeted for study in this manuscript at K_{helix} (G/E305) and H_{helix} (G/T221) are marked with arrows in red colour.

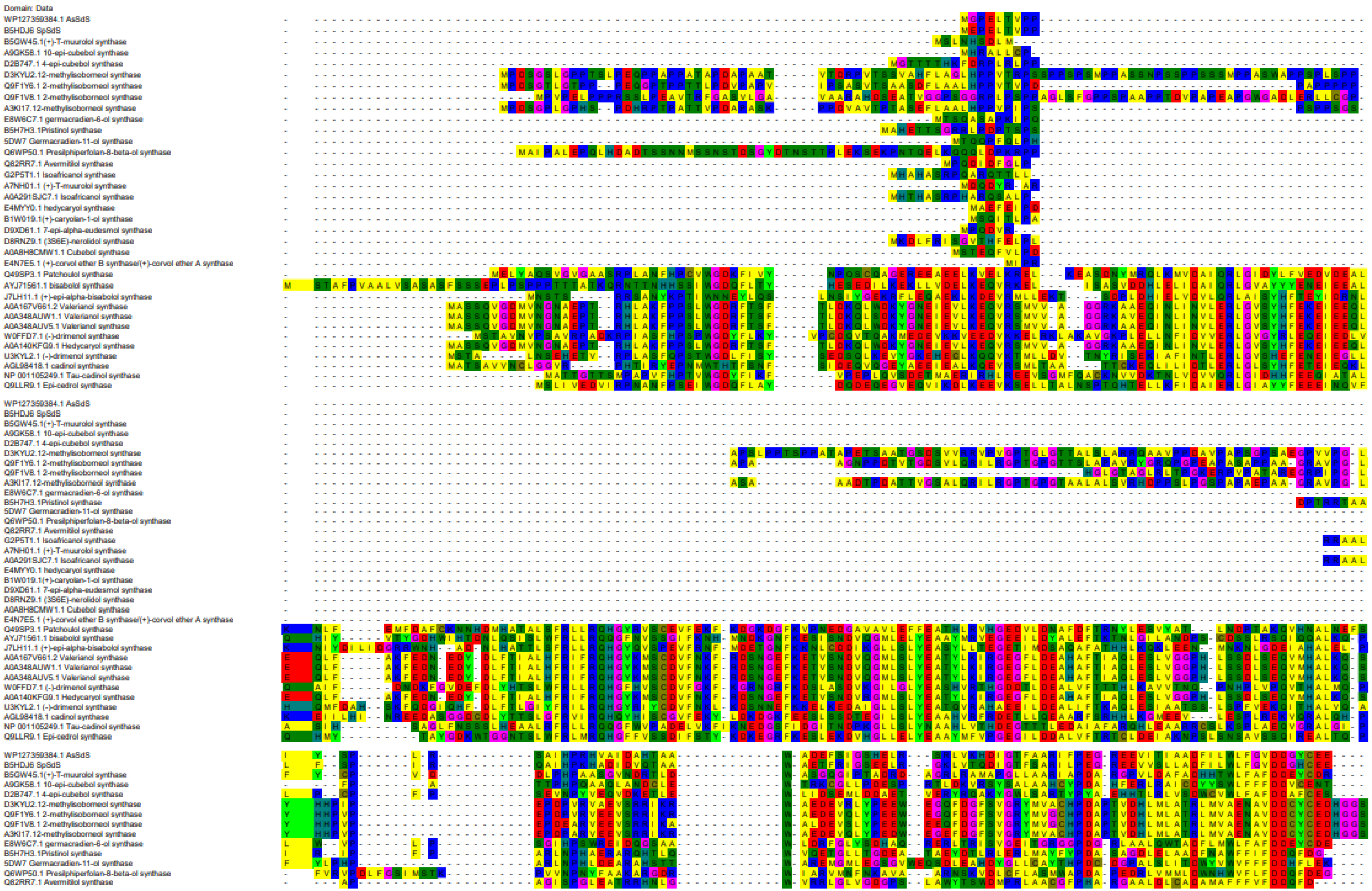
Domain: Data
WP127359384.1 AsSdS
B5HDJ6 SpSdS
B5GW45.1(+)-T-muurolol synthase
A9GK58.1 10-epi-cubebol synthase
D2B747.1 4-epi-cubebol synthase
D3KYU2.12-methylisoborneol synthase
Q9F1Y6.1 2-methylisoborneol synthase
Q9F1V8.1 2-methylisoborneol synthase
A3KI17.12-methylisoborneol synthase
E8W6C7.1 germacradien-6-ol synthase
B5H7H3.1Pristinol synthase
5DW7 Germacradien-11-ol synthase
Q6WP50.1 Presilphiperfolan-8-beta-ol synthase
Q82RR7.1 Avermitilol synthase
G2P5T1.1 Isoafricanol synthase
A7NH01.1 (+)-T-muurolol synthase
A0A291SJC7.1 Isoafricanol synthase
E4MYY0.1 hedycaryol synthase
B1W019.1(+)-caryolan-1-ol synthase
D9XD61.1 7-epi-alpha-eudesmol synthase
D8RNZ9.1 (3S6E)-nerolidol synthase
A0A8H8CMW1.1 Cubebol synthase
E4N7E5.1 (+)-corvol ether B synthase/(+)-corvol ether A synthase
Q49SP3.1 Patchoulol synthase
AYJ71561.1 bisabolol synthase
J7LH11.1 (+)-epi-alpha-bisabolol synthase
A0A167V661.2 Valerianol synthase
A0A348AUW1.1 Valerianol synthase
A0A348AUV5.1 Valerianol synthase
W0FFD7.1 (-)-drimenol synthase
A0A140KFG9.1 Hedycaryol synthase
U3KYL2.1 (-)-drimenol synthase
AGL98418.1 cadinol synthase
NP 001105249.1 Tau-cadinol synthase
Q9LLR9.1 Epi-cedrol synthase

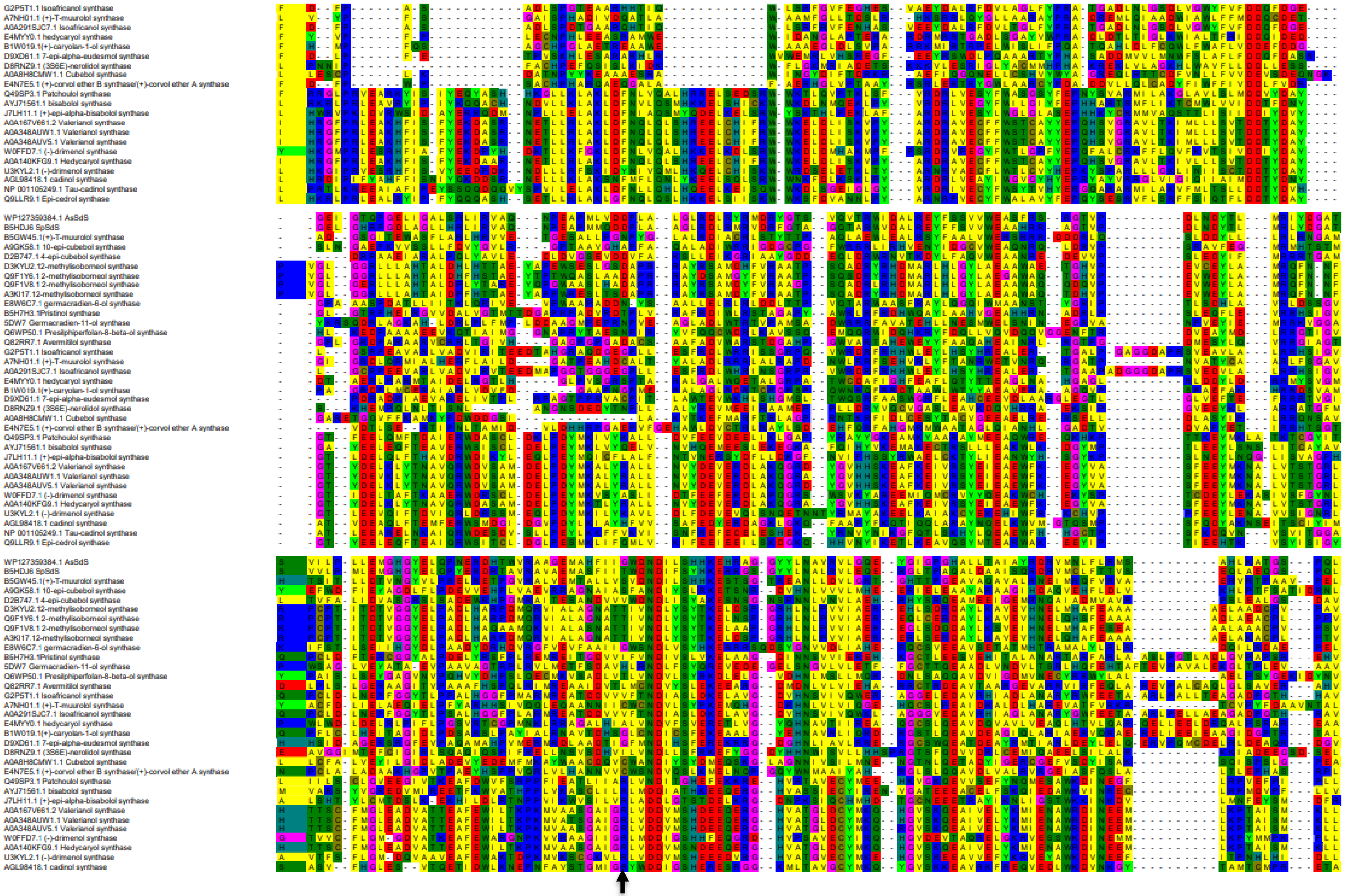

G2P5T1.1 Isoafricanol synthase
A7NH01.1 (+)-T-muurolol synthase
A0A291SJC7.1 Isoafricanol synthase
E4MYY0.1 hedycaryol synthase
B1W019.1(+)-caryolan-1-ol synthase
D9XD61.1 7-epi-alpha-eudesmol synthase
D8RNZ9.1 (3S6E)-nerolidol synthase
A0A8H8CMW1.1 Cubebol synthase
E4N7E5.1 (+)-corvol ether B synthase/(+)-corvol ether A synthase
Q49SP3.1 Patchoulol synthase
AYJ71561.1 bisabolol synthase
J7LH11.1 (+)-epi-alpha-bisabolol synthase
A0A167V661.2 Valerianol synthase
A0A348AUW1.1 Valerianol synthase
A0A348AUV5.1 Valerianol synthase
W0FFD7.1 (-)-drimenol synthase
A0A140KFG9.1 Hedycaryol synthase
U3KYL2.1 (-)-drimenol synthase
AGL98418.1 cadinol synthase
NP 001105249.1 Tau-cadinol synthase
Q9LLR9.1 Epi-cedrol synthase
WP127359384.1 AsSdS
B5HDJ6 SpSdS
B5GW45.1(+)-T-muurolol synthase
A9GK58.1 10-epi-cubebol synthase
D2B747.1 4-epi-cubebol synthase
D3KYU2.12-methylisoborneol synthase
Q9F1Y6.1 2-methylisoborneol synthase
Q9F1V8.1 2-methylisoborneol synthase
A3KI17.12-methylisoborneol synthase
E8W6C7.1 germacradien-6-ol synthase
B5H7H3.1Pristinol synthase
5DW7 Germacradien-11-ol synthase
Q6WP50.1 Presilphiperfolan-8-beta-ol synthase
Q82RR7.1 Avermitilol synthase
G2P5T1.1 Isoafricanol synthase
A7NH01.1 (+)-T-muurolol synthase
A0A291SJC7.1 Isoafricanol synthase
E4MYY0.1 hedycaryol synthase
B1W019.1(+)-caryolan-1-ol synthase
D9XD61.1 7-epi-alpha-eudesmol synthase
D8RNZ9.1 (3S6E)-nerolidol synthase
A0A8H8CMW1.1 Cubebol synthase
E4N7E5.1 (+)-corvol ether B synthase/(+)-corvol ether A synthase
Q49SP3.1 Patchoulol synthase
AYJ71561.1 bisabolol synthase
J7LH11.1 (+)-epi-alpha-bisabolol synthase
A0A167V661.2 Valerianol synthase
A0A348AUW1.1 Valerianol synthase
A0A348AUV5.1 Valerianol synthase
W0FFD7.1 (-)-drimenol synthase
A0A140KFG9.1 Hedycaryol synthase
U3KYL2.1 (-)-drimenol synthase
AGL98418.1 cadinol synthase
NP 001105249.1 Tau-cadinol synthase
Q9LLR9.1 Epi-cedrol synthase
WP127359384.1 AsSdS
B5HDJ6 SpSdS
B5GW45.1(+)-T-muurolol synthase
A9GK58.1 10-epi-cubebol synthase
D2B747.1 4-epi-cubebol synthase
D3KYU2.12-methylisoborneol synthase
Q9F1Y6.1 2-methylisoborneol synthase
Q9F1V8.1 2-methylisoborneol synthase
A3KI17.12-methylisoborneol synthase
E8W6C7.1 germacradien-6-ol synthase
B5H7H3.1Pristinol synthase
5DW7 Germacradien-11-ol synthase
Q6WP50.1 Presilphiperfolan-8-beta-ol synthase
Q82RR7.1 Avermitilol synthase
G2P5T1.1 Isoafricanol synthase
A7NH01.1 (+)-T-muurolol synthase
A0A291SJC7.1 Isoafricanol synthase
E4MYY0.1 hedycaryol synthase
B1W019.1(+)-caryolan-1-ol synthase
D9XD61.1 7-epi-alpha-eudesmol synthase
D8RNZ9.1 (3S6E)-nerolidol synthase
A0A8H8CMW1.1 Cubebol synthase
E4N7E5.1 (+)-corvol ether B synthase/(+)-corvol ether A synthase
Q49SP3.1 Patchoulol synthase
AYJ71561.1 bisabolol synthase
J7LH11.1 (+)-epi-alpha-bisabolol synthase
A0A167V661.2 Valerianol synthase
A0A348AUW1.1 Valerianol synthase
A0A348AUV5.1 Valerianol synthase
W0FFD7.1 (-)-drimenol synthase
A0A140KFG9.1 Hedycaryol synthase
U3KYL2.1 (-)-drimenol synthase
AGL98418.1 cadinol synthase

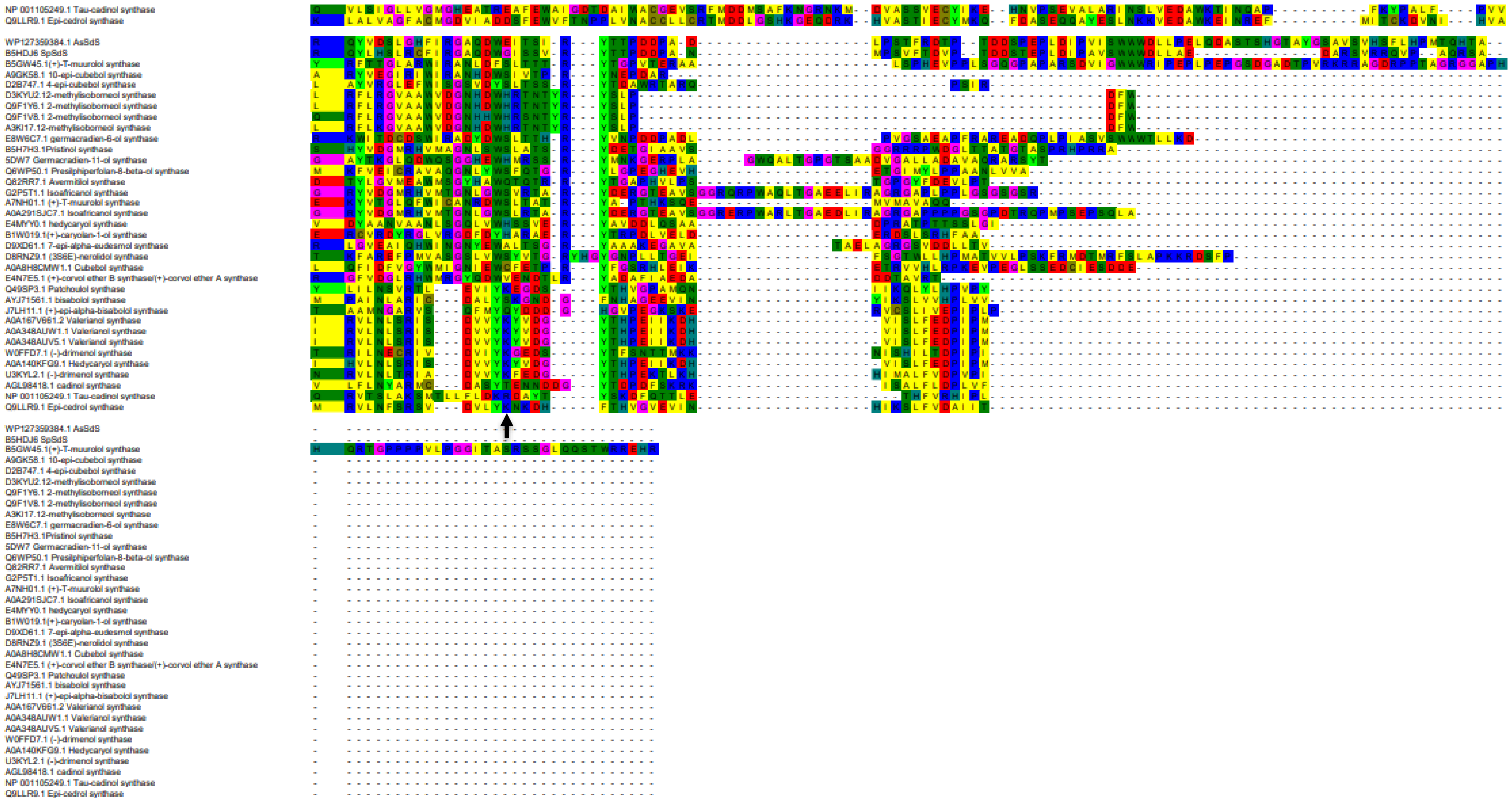

Figure S2: Multiple sequence alignment of functionally characterised hydroxylating sesquiterpene synthases along with selinadiene synthases using MEGA11.

11. Total ion chromatograms (TICs)

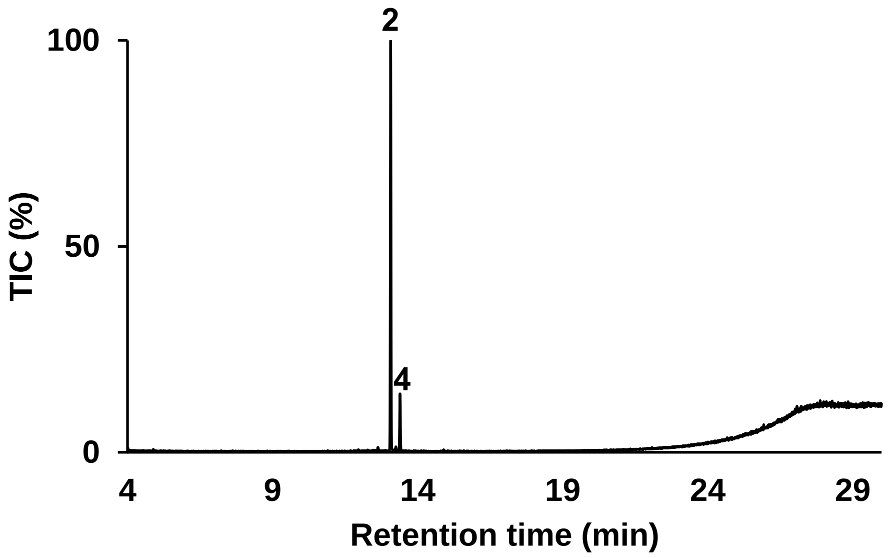

Figure S3. TIC of pentane extractable products arising from the incubation of SpSdS with (2*E*,6*E*)-FDP (**1**), producing selina-4(15),7(11)-diene (**2**) as the major product along with germacrene B (**4**) as a minor product.

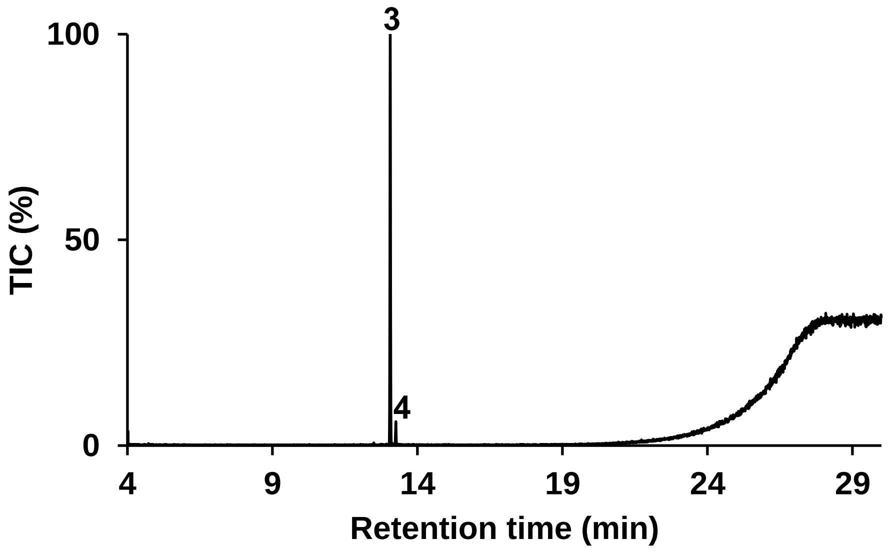

Figure S4. TIC of pentane extractable product arising from the incubation of AsSdS with (2*E*,6*E*)-FDP (**1**), producing selina-3,7(11)-diene (**3**) as the major product along with minor quantities of germacrene B (**4**).

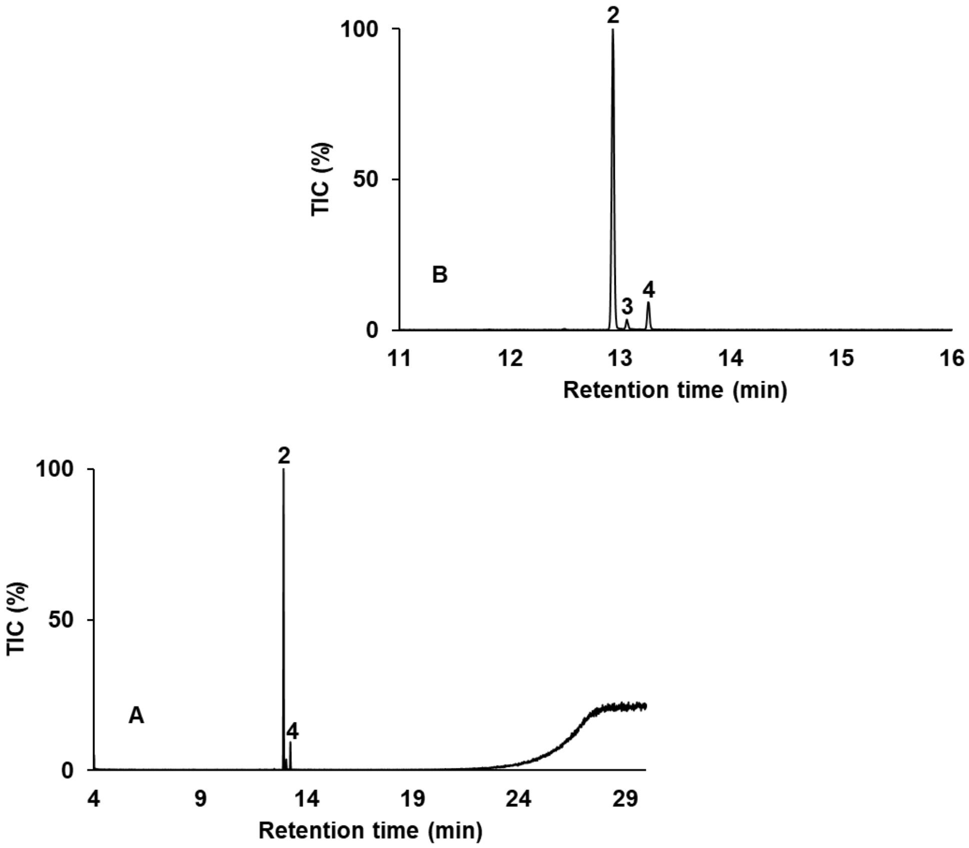

Figure S5. TIC of pentane extractable product arising from the incubation of AsSdS E305G with (2*E*,6*E*)-FDP (**1**), producing selina-4(15),7(11)-diene (**2**) as the major product along with minor quantities of selina-3,7(11)-diene (**3**) and germacrene B. **A)** Full chromatogram. **B)** Zoomed chromatogram for improved clarity.

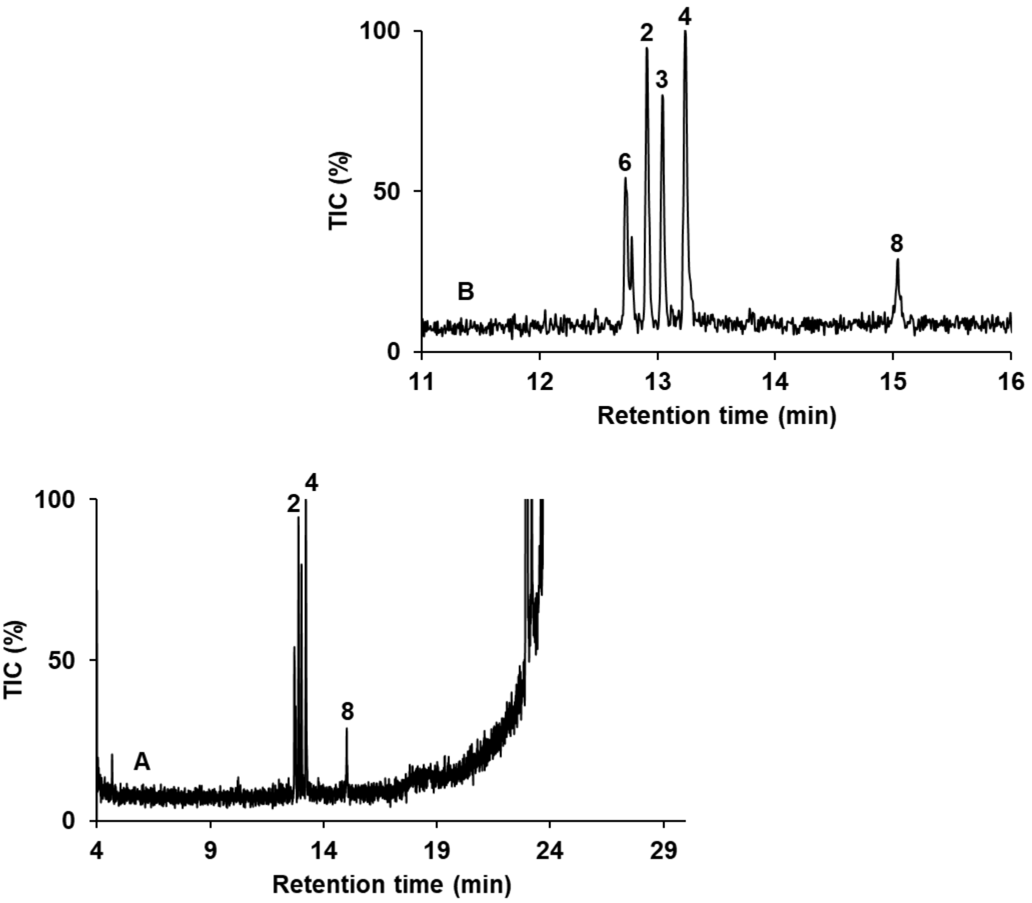

Figure S6. TIC of pentane extractable product arising from the incubation of AsSdS G221T with (2*E*,6*E*)-FDP (**1**), producing germacrene B (**4**) as a major product with reduced level of selina-3,7(11)-diene (**3**) along with the formation of selina-4(15),7(11)-diene (**2**) and minor quantities of selin-7(11)-en-4-ol (**8**). **A)** Full chromatogram. **B)** Zoomed chromatogram for improved clarity.

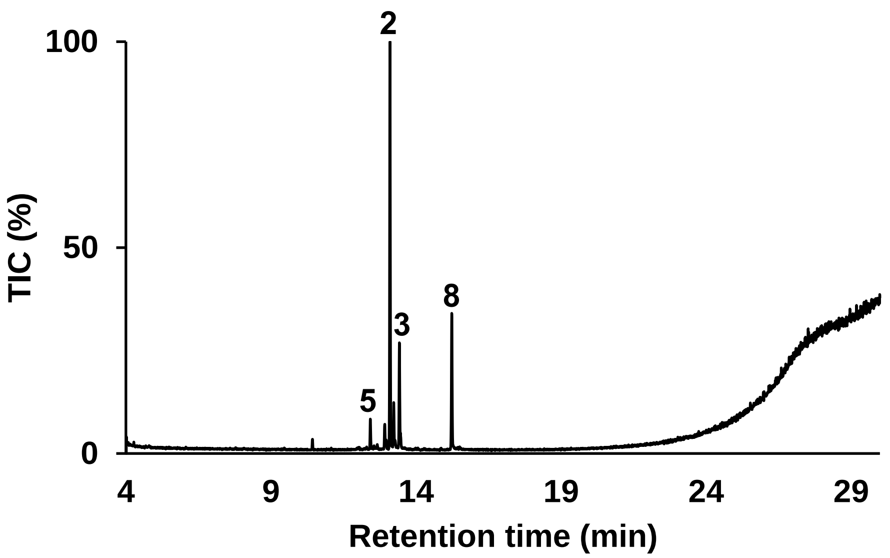

Figure S7. TIC of pentane extractable product arising from the incubation of SpSdS G305E[3] with (2*E*,6*E*)-FDP (**1**), producing selina-4(15),7(11)-diene (**2**) as a major product along with hydroxylated sesquiterpene selin-7(11)-en-4-ol (**8**) and traces of selina-3,7(11)-diene (**3**), germacrene B (**4**) and δ-selinene (**5**).

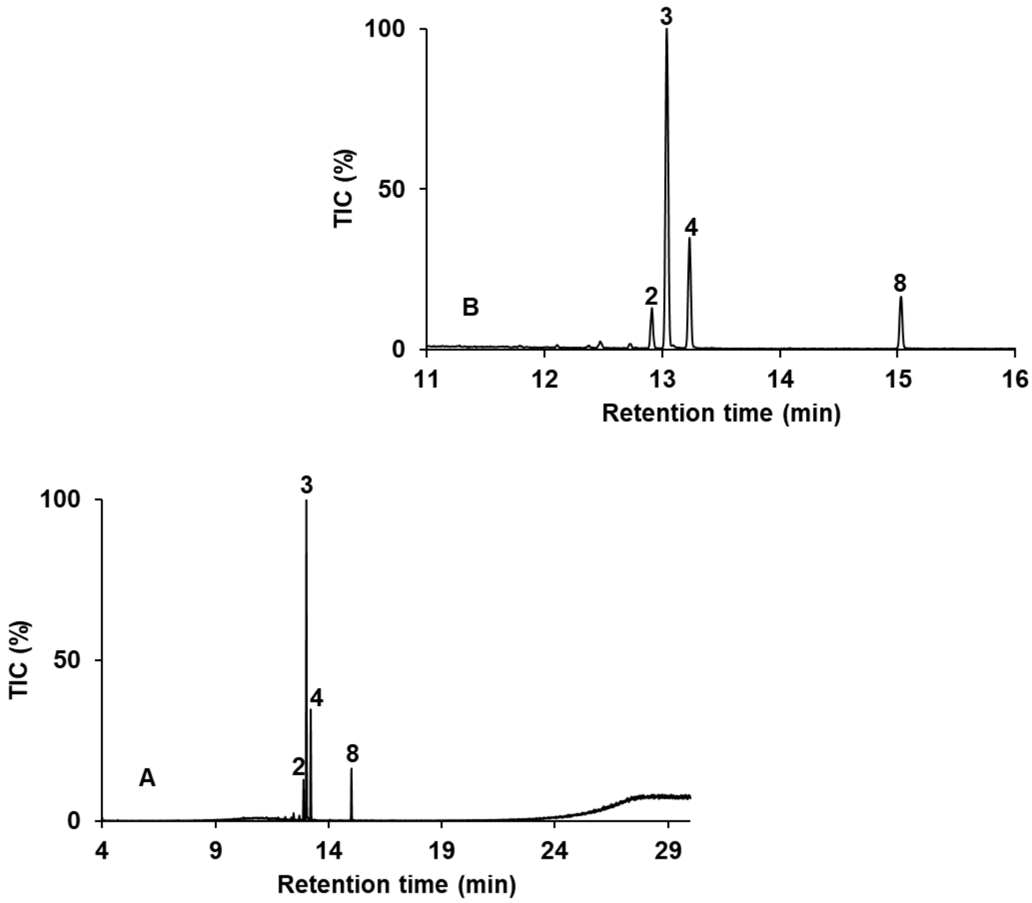

Figure S8. TIC of pentane extractable product arising from the incubation SpSdS G305E + T221G with (2*E*,6*E*)-FDP (**1**), producing selina-3,7(11)-diene (**3**) as a major product along with minor quantities of selina-4(15),7(11)-diene (**2**) and reduced level of selin-7(11)-en-4-ol (**8**). **A)** Full chromatogram. **B)** Zoomed chromatogram for improved clarity.

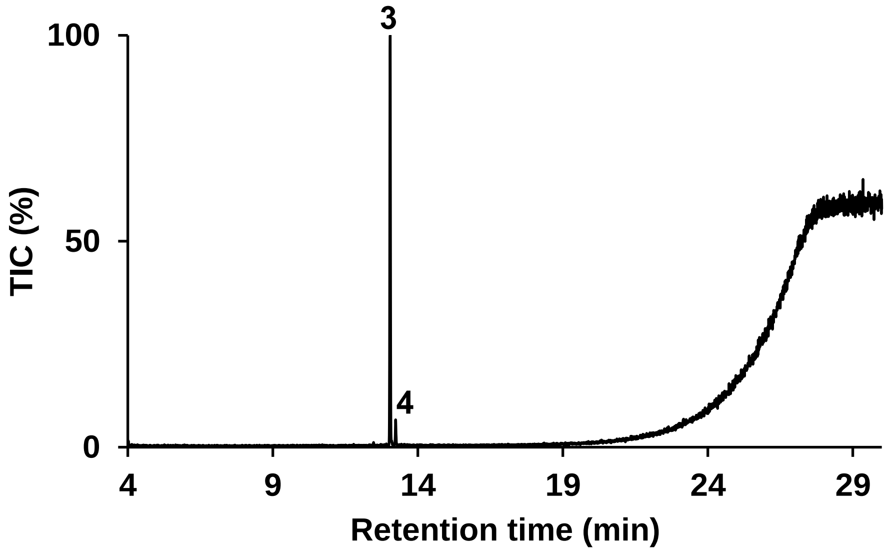

Figure S9. Preparative scale incubation of AsSdS for NMR spectroscopic characterisation. TIC of pentane extractable product arising from incubation of AsSdS with (2*E*,6*E*)-FDP (**1**). The major sesquiterpene fraction was confirmed as selina-3,7(11)-diene (**3**) by NMR spectroscopy and comparing the observed spectral with data reported in the literature.[9–11]

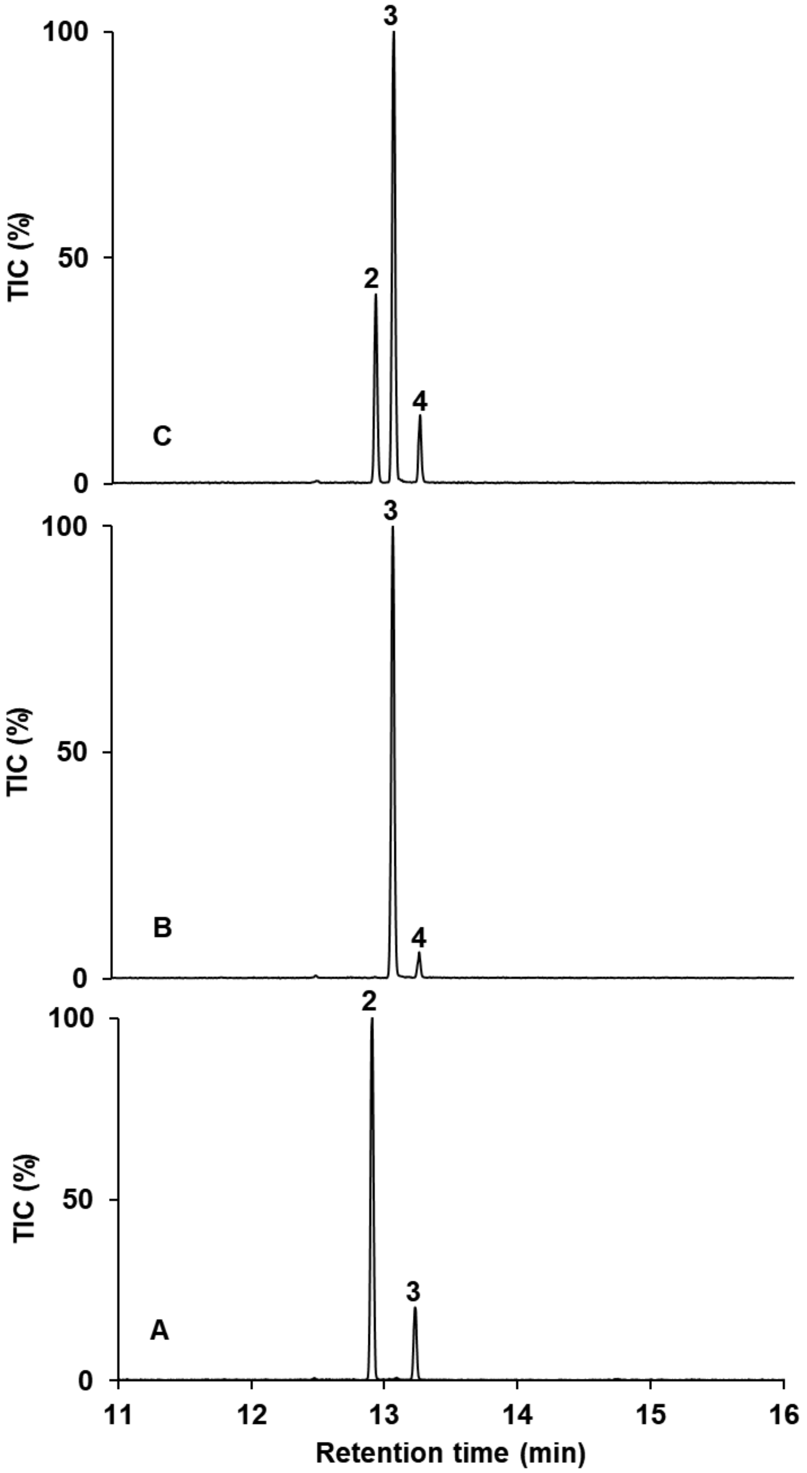

Figure S10. Co-injection of AsSdS and SpSdS assay samples. **A)** TIC of pentane extractable product arising from incubation of SpSdS with (2*E*,6*E*)-FDP (**1**) producing selina-4(15),7(11)-diene (**2**) as a major product along with germacrene B (**4**) as a minor product. **B)** TIC of pentane extractable product arising from incubation of AsSdS with (2*E*,6*E*)-FDP (**1**) producing selina-3,7(11)-diene (**3**) as the major product along with minor quantities of germacrene B (**4**). **C)** TIC of co-injection of pentane extractable product arising from the incubation of SpSdS and AsSdS (2*E*,6*E*)-FDP (**1**).

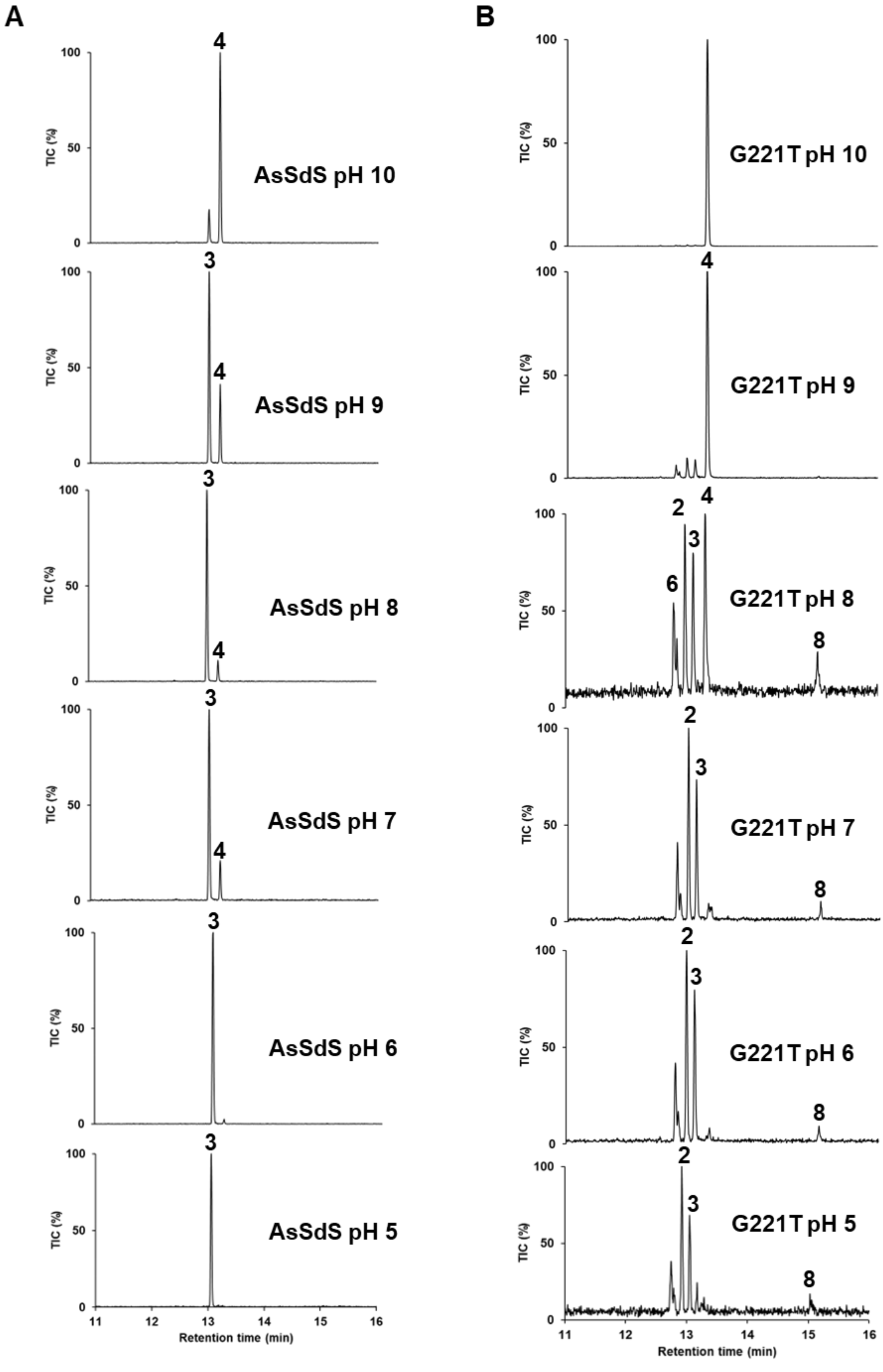

Figure S11: pH studies of AsSdS wild-type and AsSdS G221T. **A)** TICs of pentane extractable product arising from incubation of AsSdS with (2*E*,6*E*)-FDP (**1**) producing selina-3,7(11)-diene (**3**) as a major product at lower pH and

germacrene B (**4**) as a minor product at higher pH. **B)** TICs of pentane extractable product arising from incubation of AsSdS G221T with (2*E*,6*E*)-FDP (**1**) producing selinadienes: selina-4(15),7(11)-diene (**2**), selina-3,7(11)-diene (**3**) at lower pH with small percentage of selin-7(11)-en-4-ol (**8**) and germacrene B (**4**) as major product at higher pH.

12. GCMS Mass Spectra

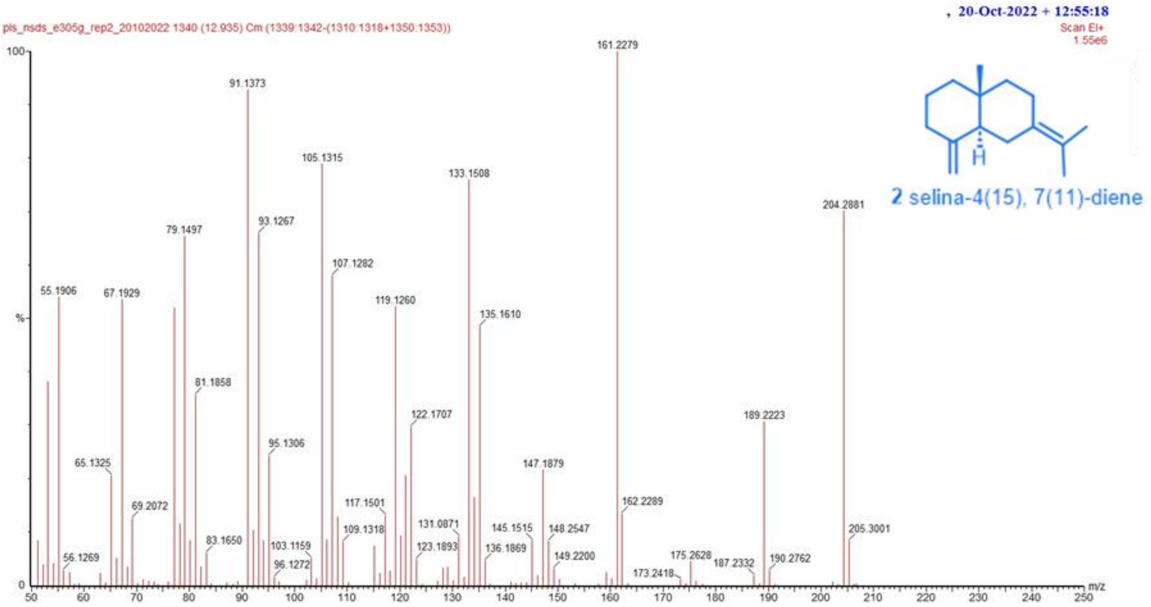

Figure 12: EI$^+$ Mass spectrum of the compound eluting at 13.06 min in the gas-chromatogram (selina-4(15),7(11)-diene, **2**) from the incubation of (2*E*,6*E*)-FDP (**1**) with SpSdS and AsSdS E305G.

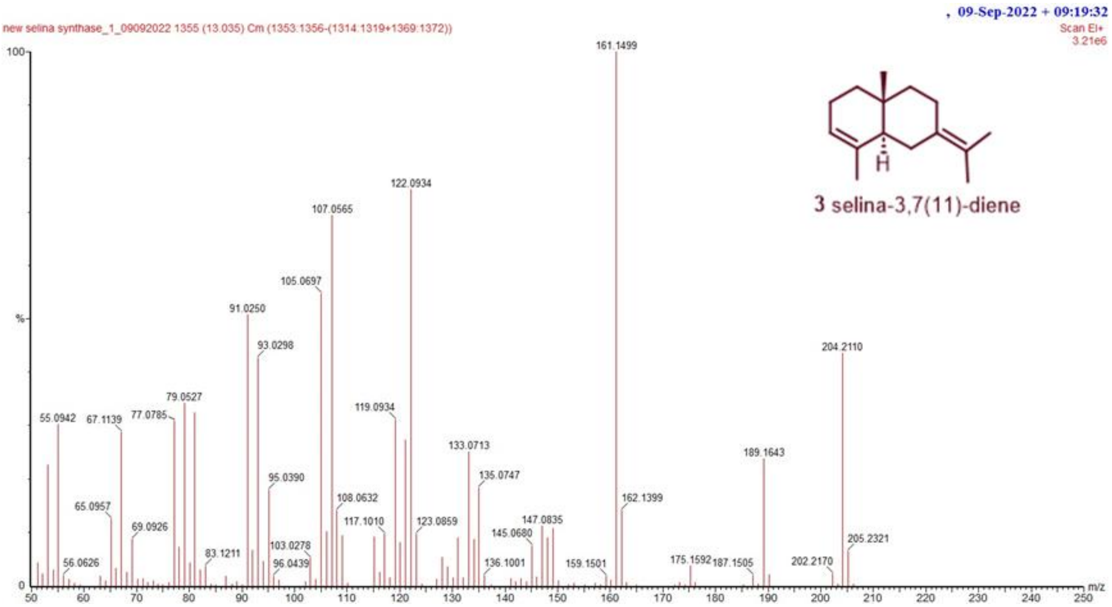

Figure 13: EI$^+$ Mass spectrum of the compound eluting at 13.20 min in the gas-chromatogram (selina-3,7(11)-diene, **3**) from the incubation of (2*E*,6*E*)-FDP (**1**) with AsSdS, AsSdS G221T, AsSdS E305G, SpSdS G305E and SpSdS G305E + T221G.

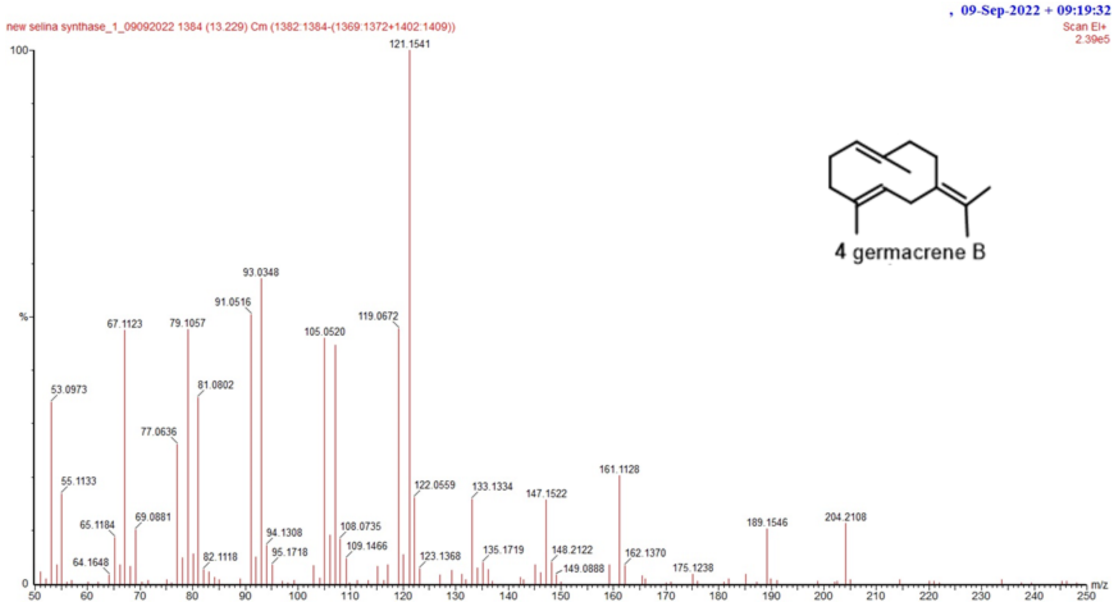

Figure 14: EI$^+$ Mass spectrum of the compound eluting at 13.38 min in the gas-chromatogram (germacrene B, **4**) from the incubation of (2*E*,6*E*)-FDP (**1**) with SpSdS, AsSdS and other variants.

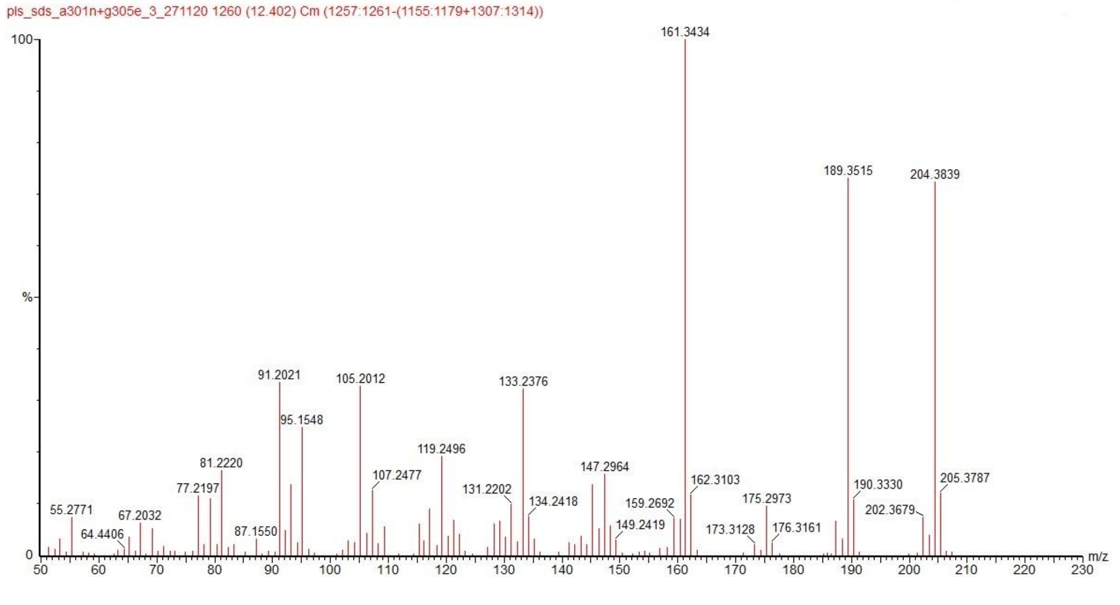

Figure 15: EI$^+$ Mass spectrum of the compound eluting at 12.37 min in the gas-chromatogram (δ-selinene, **5**) from the incubation of (2*E*,6*E*)-FDP (**1**) with SpSdS G305E.

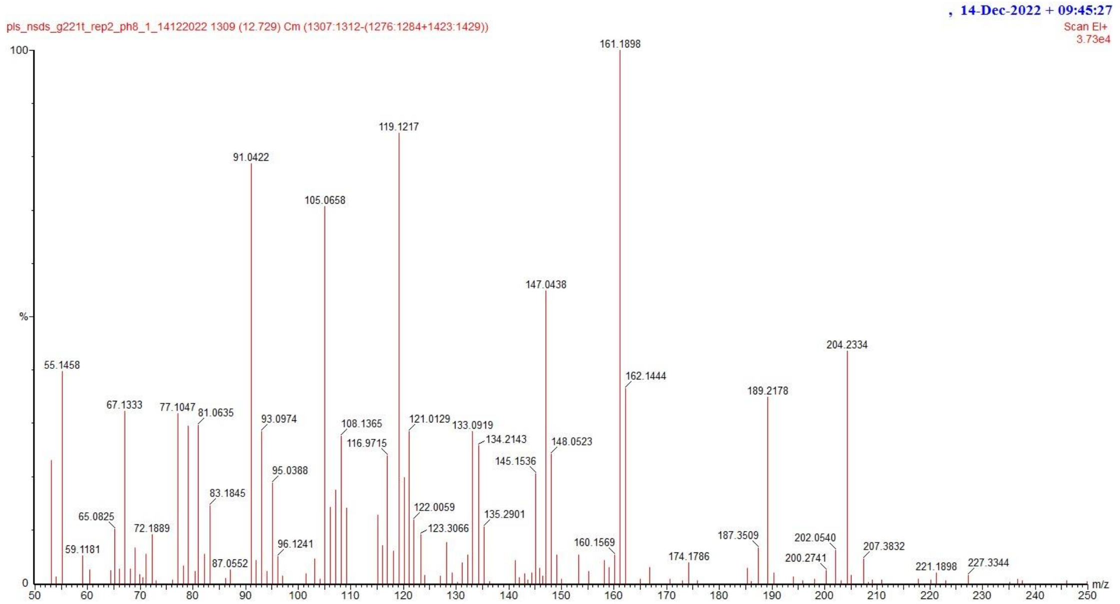

Figure 16: EI$^+$ Mass spectrum of the compound eluting at 12.73 min in the gas-chromatogram (uncharacterised sesquiterpene, **6**) from the incubation of (2*E*,6*E*)-FDP (**1**) with AsSdS G221T.

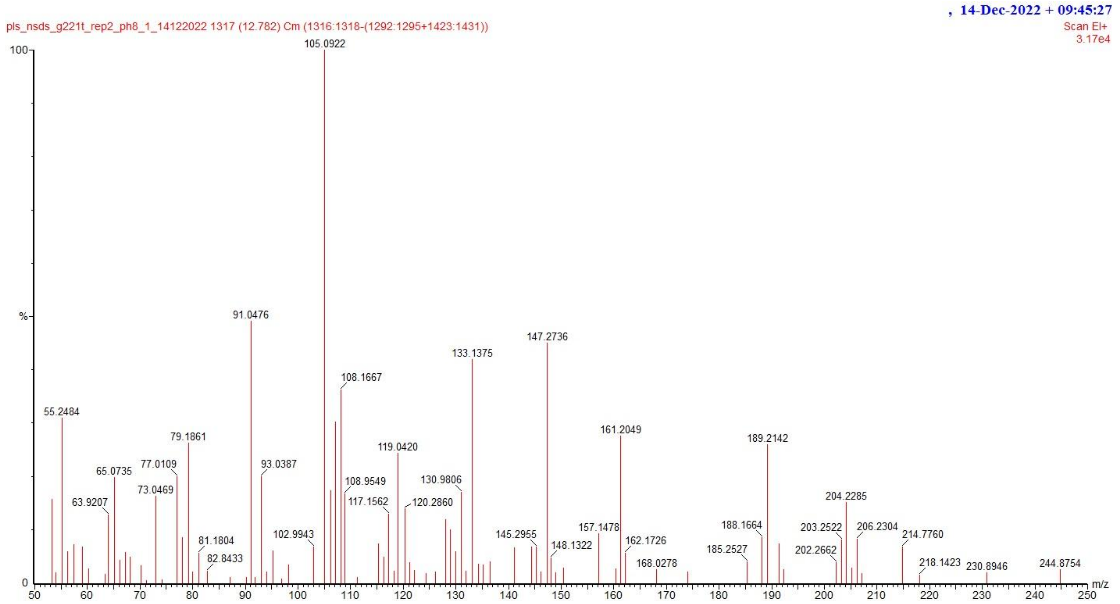

Figure 17: EI$^+$ Mass spectrum of the compound eluting at 12.88 min in the gas-chromatogram (uncharacterised sesquiterpene, **7**) from the incubation of (2*E*,6*E*)-FDP (**1**) with SpSdS G305E and AsSdS G221T.

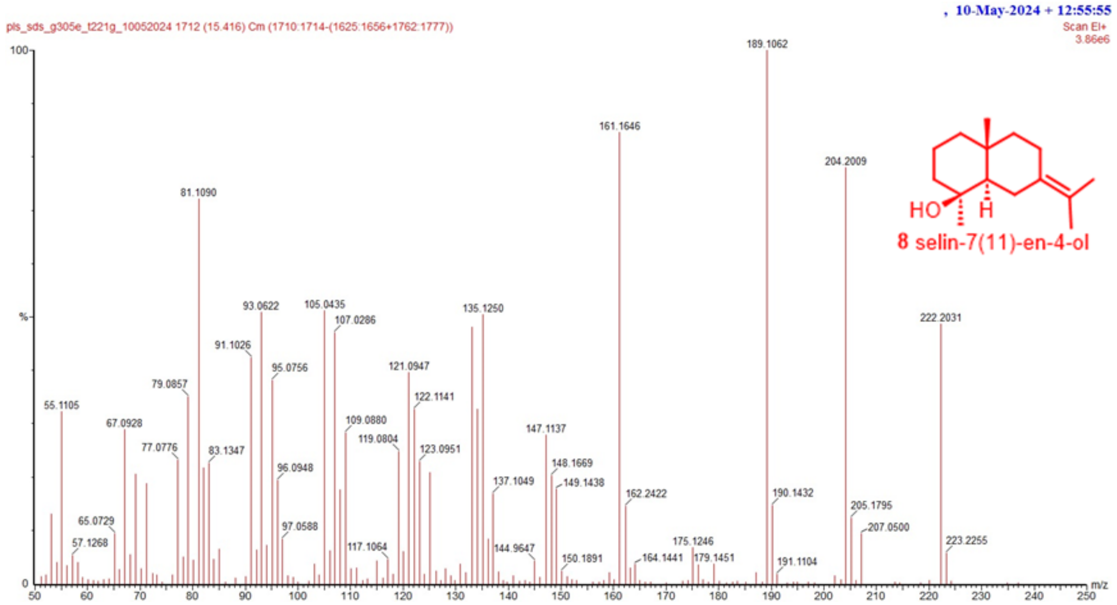

Figure 18: EI$^+$ Mass spectrum of the compound eluting at 15.17 min in the gas-chromatogram (selin-7(11)-en-4-ol, **8**) from the incubation of (2*E*,6*E*)-FDP (**1**) with SpSdS G305E, AsSdS G221T and SpSdS G305E+T221G.

13. Kinetic Data

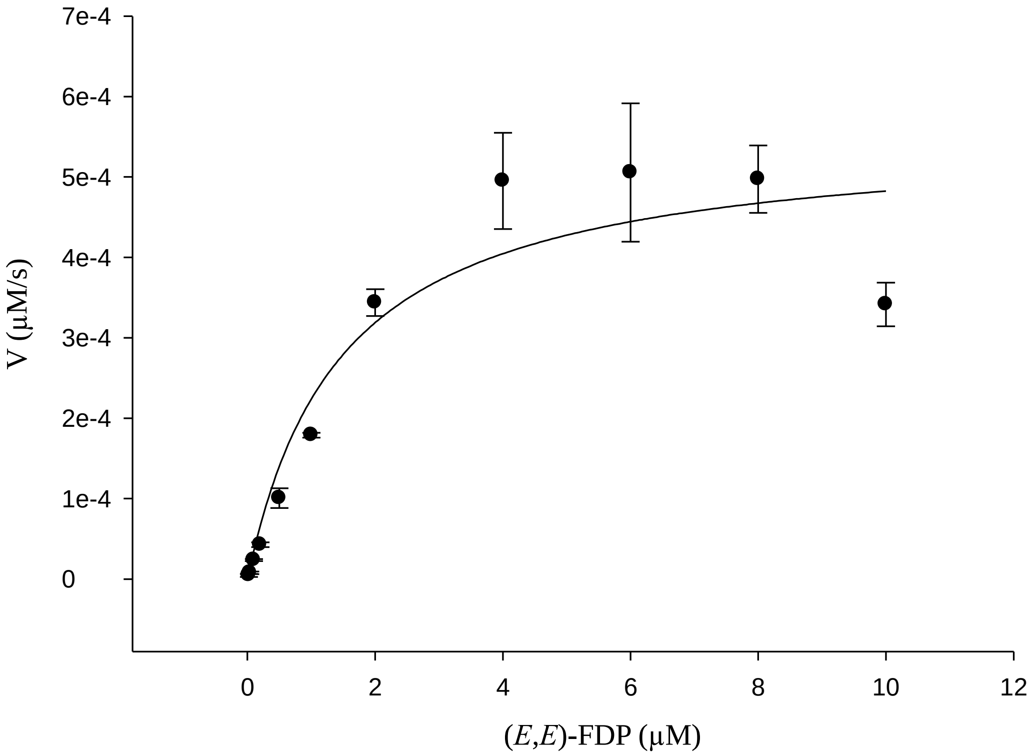

Figure S19: Representative Michaelis-Menten plot for the conversion of [1-^{3}H]-FDP by AsSdS.

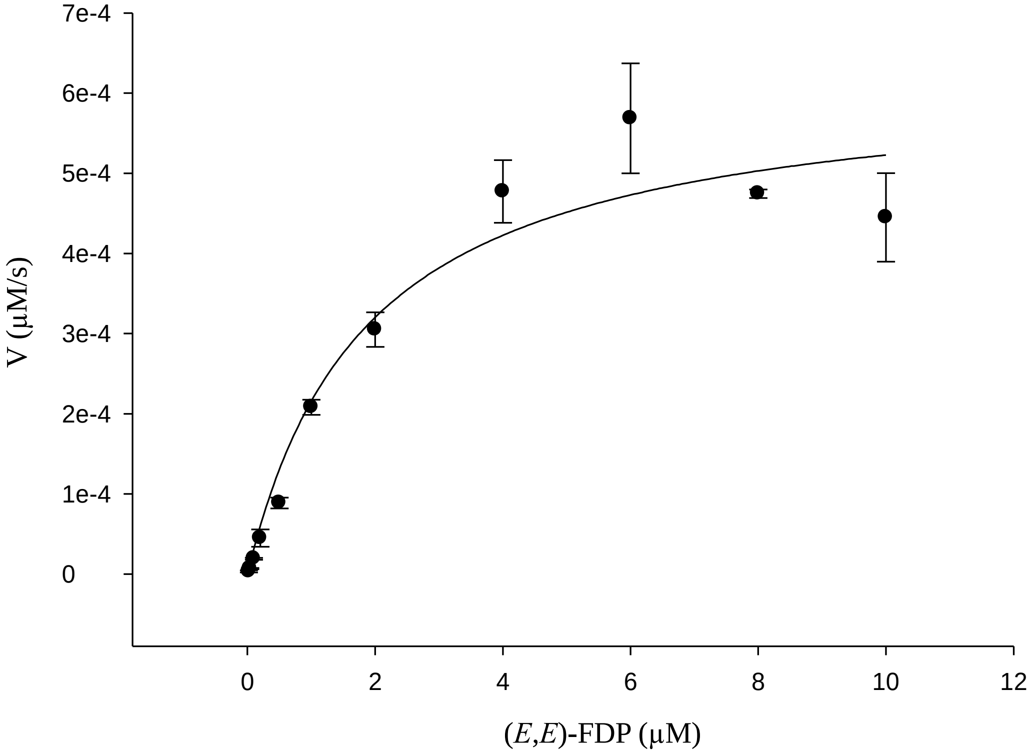

Figure S20: Representative Michaelis-Menten plot for the conversion of [1-^{3}H]-FDP by AsSdS E305G.

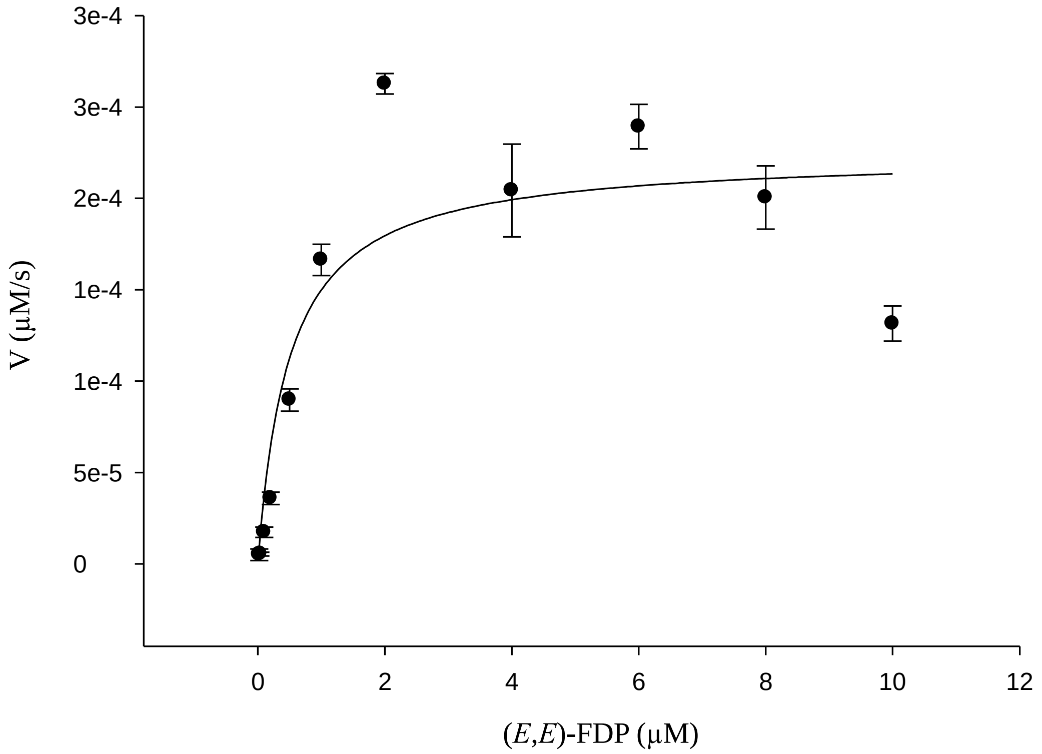

Figure S21: Representative Michaelis-Menten plot for the conversion of [1-^{3}H]-FDP by AsSdS G221T.

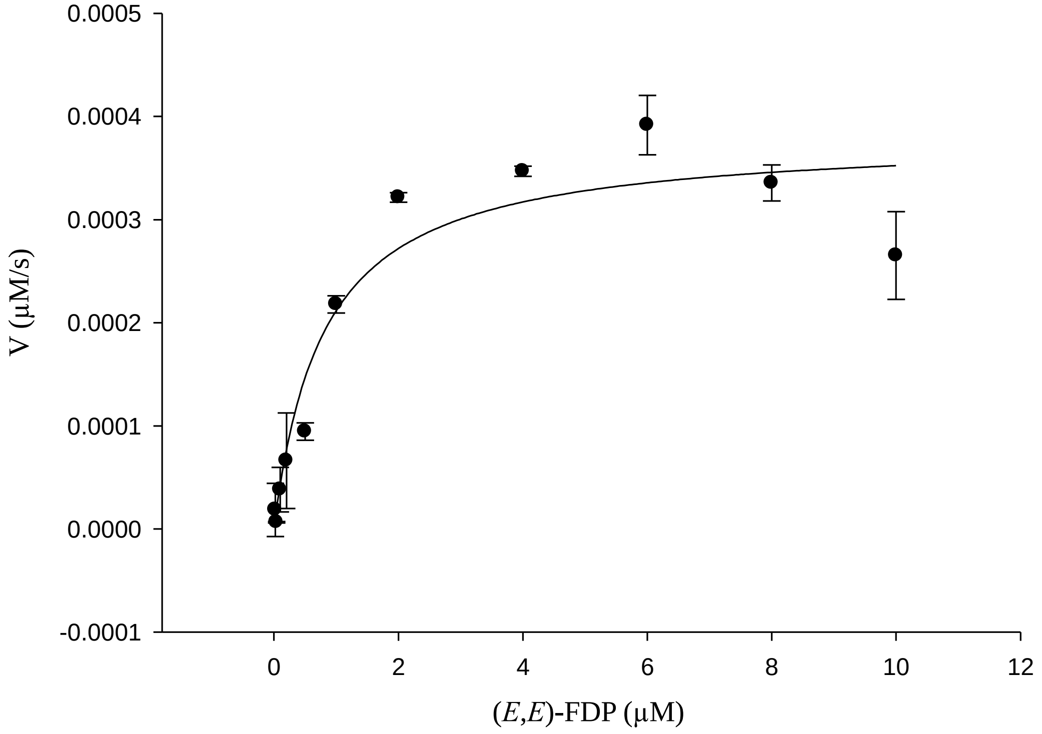

Figure S22: Representative Michaelis-Menten plot for the conversion of [1-^{3}H]-FDP by SpSdS G305E +T221G.

14. NMR Spectra

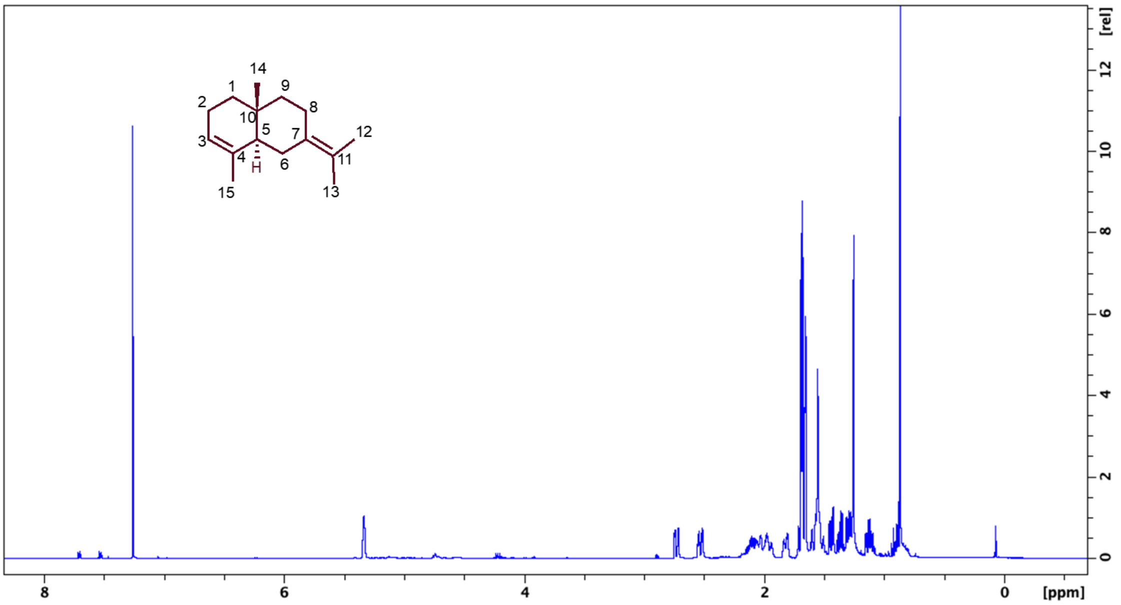

Figure 23A: ^{1}H NMR spectrum (500 MHz, $CDCl_3$, 298K) of selina-3,7(11)-diene (**3**) full.

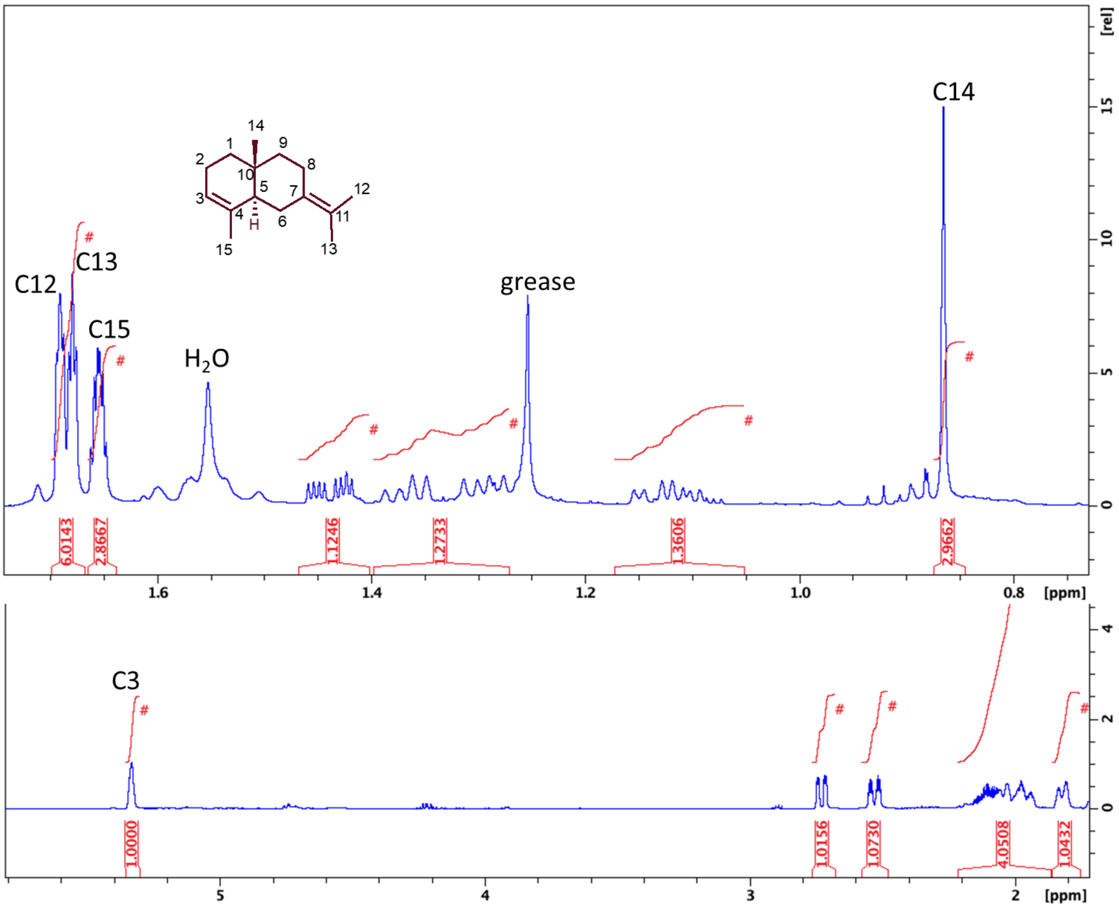

Figure 23B: ^{1}H NMR spectrum (500 MHz, $CDCl_3$, 298K) of selina-3,7(11)-diene (**3**) expansions.

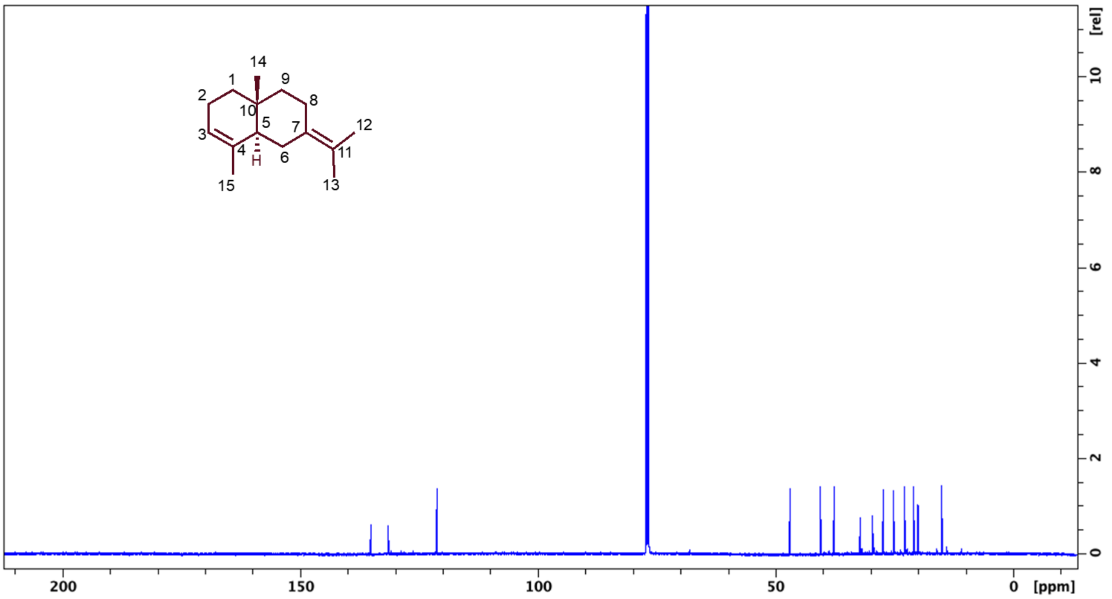

Figure 24A: ^{13}C NMR spectrum (125 MHz, $CDCl_3$, 298K) of selina-3,7(11)-diene (**3**). Peak at 29.71 represents the contamination of grease.

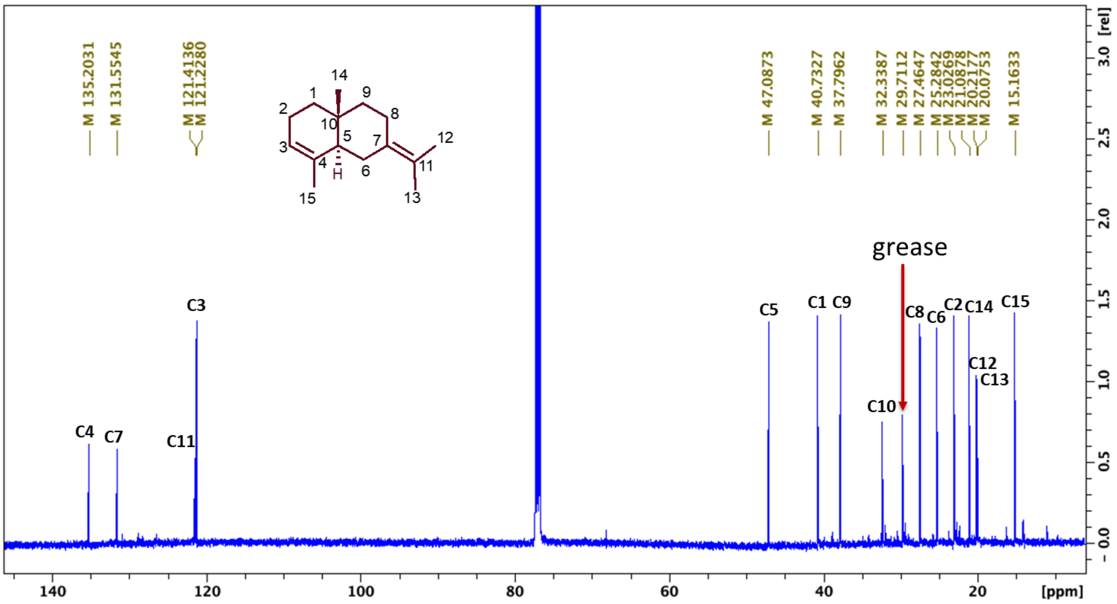

Figure 24B: Expansion of the ^{13}C NMR spectrum (125 MHz, $CDCl_3$, 298K) of selina-3,7(11)-diene (**3**). Peak at δ_C = 29.71 represents a small contamination of grease.

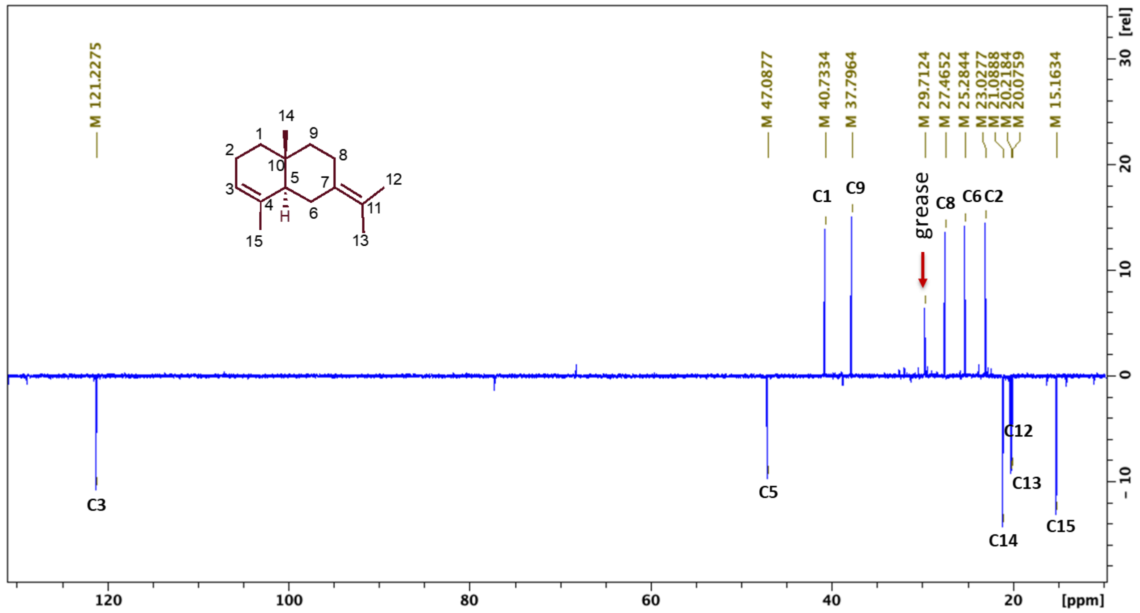

Figure 25: DEPT 135 NMR spectrum (125 MHz, $CDCl_3$, 298K) of selina-3,7(11)-diene (**3**). Peak at δ_C = 29.71 represent a small contamination of grease.

15. Computational image

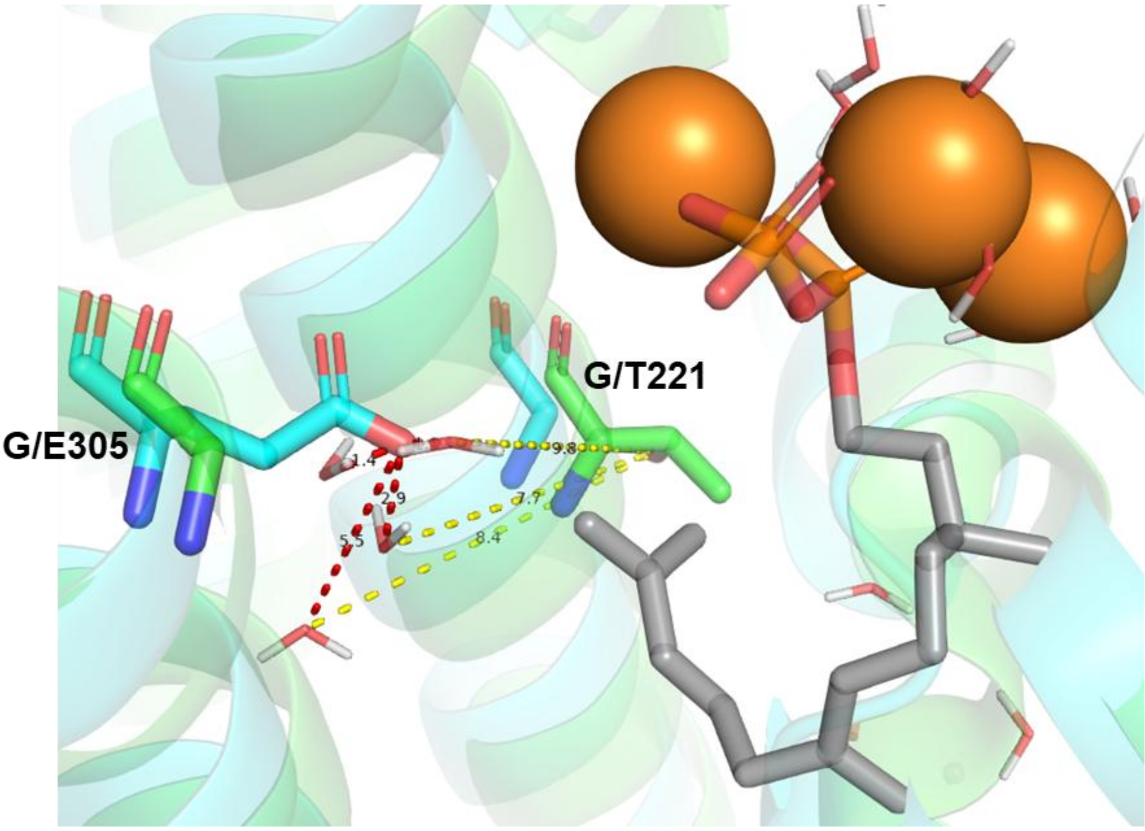

Figure S26: Measurement of distances (Å) from E/G 305 at K_{helix} and G/T221 in H_{helix} in AsSdS and SpSdS (cyan: AsSdS, green: SpSdS) with trapped water molecules indicating that could create a bridge required for quenching the final carbocation to form selin-7(11)-en-4-ol (**8**) by selinadiene synthase variants containing E305 at K_{helix} and T221 at H_{helix}. Trapped water molecules are highted as stick in red colour. AsSdS homology model was overlaid with crystal structure reported for SpSdS (pdb: 4OKZ).

16. SDS-PAGE analysis

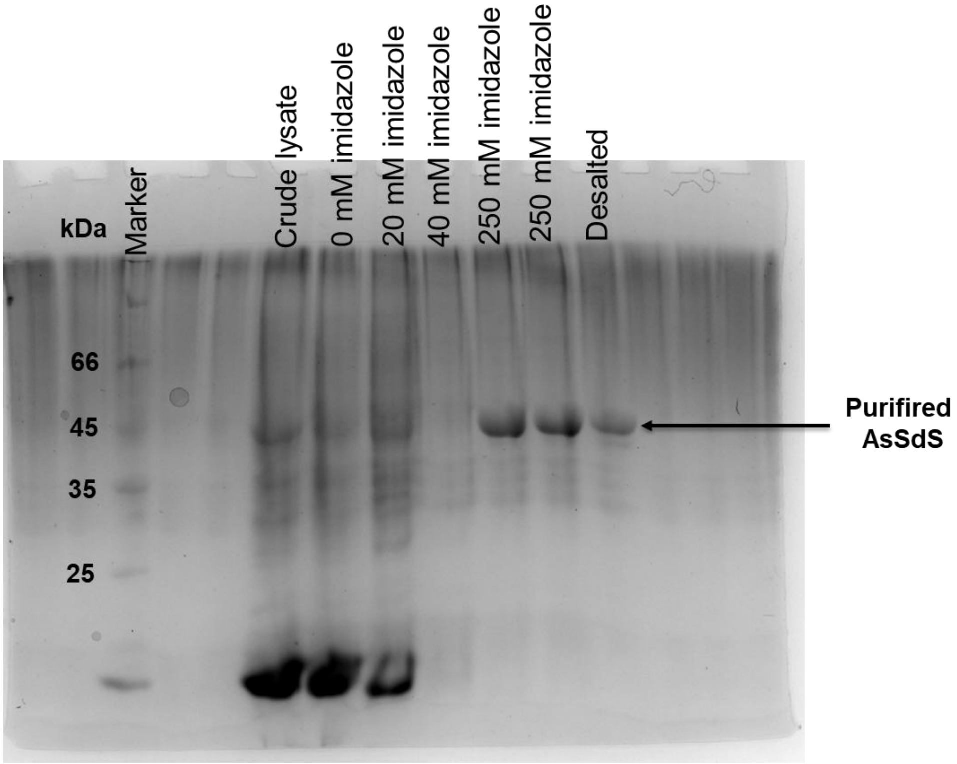

Figure S27: SDS-PAGE analysis of purification of AsSdS using Ni-NTA column chromatography.

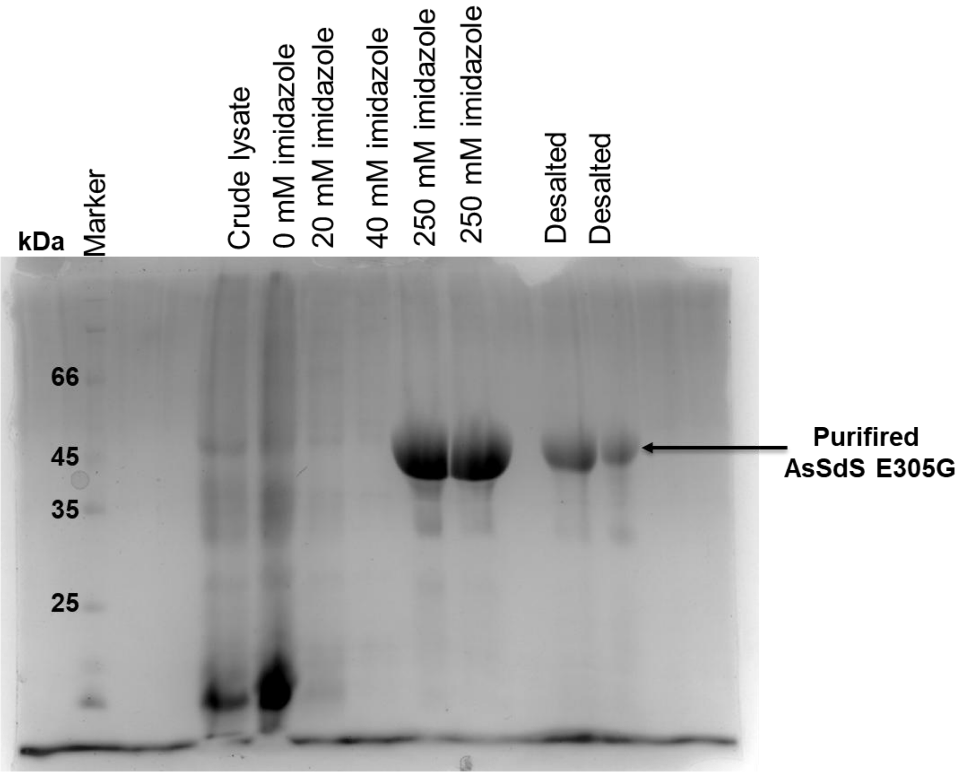

Figure S28: SDS-PAGE analysis of purification of AsSdS E305G using Ni-NTA column chromatography.

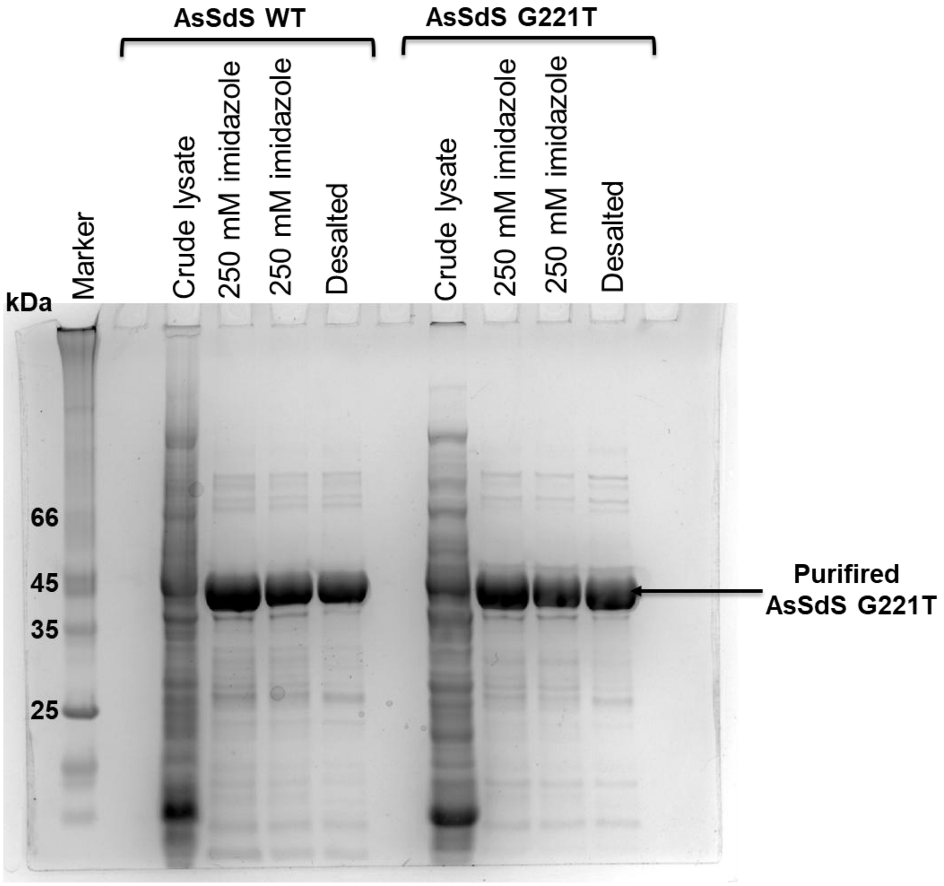

Figure S29: SDS-PAGE analysis of purification of AsSdS wild-type and AsSdS variant G221T using Ni-NTA column chromatography.

16. References

[1] P. Rabe, J. S. Dickschat, *Angew Chem Int Ed* **2013**, *52*, 1810–1812.

[2] V. J. Davisson, A. B. Woodside, T. R. Neal, K. E. Stremler, M. Muehlbacher, C. D. Poulter, *J Org Chem* **1986**, *51*, 4768–4779.

[3] P. L. Srivastava, S. T. Johns, A. Voice, K. Morley, A. M. Escorcia, D. J. Miller, R. K. Allemann, M. W. van der Kamp, *ACS Catal* **2024**, *14,* 11034–11043.

[4] K. Tamura, G. Stecher, S. Kumar, *Mol Biol Evol* **2021**, *38*, 3022–3027.

[5] M. Biasini, S. Bienert, A. Waterhouse, K. Arnold, G. Studer, T. Schmidt, F. Kiefer, T. G. Cassarino, M. Bertoni, L. Bordoli, T. Schwede *Nucleic Acids Res* **2014**, *42*, 252–258.

[6] P. Baer, P. Rabe, K. Fischer, C. A. Citron, T. A. Klapschinski, M. Groll, J. S. Dickschat, *Angew Chem Int Ed* **2014**, *53*, 7652–7656.

[7] M. M. Bradford, *Anal Biochem* **1976**, *72*, 248–254.

[8] D. J. Grundy, M. Chen, V. González, S. Leoni, D. J. Miller, D. W. Christianson, R. K. Allemann, *Biochemistry* **2016**, *55*, 2112–2121.

[9] T. Chou, S. Leea, N. Y. Sinica, *Tetrahedron* **1989**, *45*, 4113–4124.

[10] J. yu Liang, C. xue You, S. shan Guo, W. juan Zhang, Y. Li, Z. feng Geng, C. fang Wang, S. shan Du, Z. wei Deng, J. Zhang, *Ind Crops Prod* **2016**, *79*, 267–273.

[11] B. Demirci, G. K. Yasdikcioğlu, K. H. C. Başer, *Turk J Chem* **2013**, *37*, 917–926.
